# The receptor tyrosine kinase Ror is required for dendrite regeneration in *Drosophila* neurons

**DOI:** 10.1371/journal.pbio.3000657

**Published:** 2020-03-12

**Authors:** Derek M. R. Nye, Richard M. Albertson, Alexis T. Weiner, J. Ian Hertzler, Matthew Shorey, Deborah C. I. Goberdhan, Clive Wilson, Kevin A. Janes, Melissa M. Rolls

**Affiliations:** 1 Biochemistry and Molecular Biology and the Huck Institutes of the Life Sciences, The Pennsylvania State University, University Park, Pennsylvania, United States of America; 2 MSTP Program, Milton S. Hershey College of Medicine, Hershey, Pennsylvania, United States of America; 3 Physiology, Anatomy and Genetics, University of Oxford, Oxford, United Kingdom; 4 Biomedical Engineering, University of Virginia, Charlottesville, Virginia, United States of America; Utrecht University, NETHERLANDS

## Abstract

While many regulators of axon regeneration have been identified, very little is known about mechanisms that allow dendrites to regenerate after injury. Using a *Drosophila* model of dendrite regeneration, we performed a candidate screen of receptor tyrosine kinases (RTKs) and found a requirement for RTK-like orphan receptor (Ror). We confirmed that Ror was required for regeneration in two different neuron types using RNA interference (RNAi) and mutants. Ror was not required for axon regeneration or normal dendrite development, suggesting a specific role in dendrite regeneration. Ror can act as a Wnt coreceptor with frizzleds (fzs) in other contexts, so we tested the involvement of Wnt signaling proteins in dendrite regeneration. We found that knockdown of fz, dishevelled (dsh), Axin, and gilgamesh (gish) also reduced dendrite regeneration. Moreover, Ror was required to position dsh and Axin in dendrites. We recently found that Wnt signaling proteins, including dsh and Axin, localize microtubule nucleation machinery in dendrites. We therefore hypothesized that Ror may act by regulating microtubule nucleation at baseline and during dendrite regeneration. Consistent with this hypothesis, localization of the core nucleation protein γTubulin was reduced in Ror RNAi neurons, and this effect was strongest during dendrite regeneration. In addition, dendrite regeneration was sensitive to partial reduction of γTubulin. We conclude that Ror promotes dendrite regeneration as part of a Wnt signaling pathway that regulates dendritic microtubule nucleation.

## Abstract 2

This study identifies the receptor tyrosine kinase Ror as a player in a Wnt signaling pathway that regulates dendritic microtubules; although dendrite development is normal in animals that lack Ror, the poorly understood process of dendrite regeneration is compromised.

## Introduction

Lifelong nervous system function requires maintenance of axons and dendrites so they can signal over long distances. Long, thin axons can be damaged by trauma and neurodegenerative disease. Rather than replacement of damaged neurons, which does not occur in most regions of the adult nervous system, axons are regrown after injury. This process of axon regeneration has been the subject of intense investigation in multiple model systems for decades [1, 2]. Dendrites can also be damaged by adverse events, but whether their regeneration contributes to long-term nervous system function is not known.

Several different types of trauma and stress have been shown to damage dendrites. During ischemia, brief disruption of blood flow leads to transient dendrite beading [3–5], and repeated rounds of beading lead to dendrite loss [6]. Seizures also lead to dendrite beading and degeneration [7]. Dendrites can experience damage during traumatic brain injury (TBI) [8–10], and dendrite degeneration has been observed in neurodegenerative disease [11–13]. It has also been shown that dendrites simplify after axon damage [14–17]. In the case of retraction after axon injury, dendrites can re-elaborate if the axon reaches a target [15]. In vertebrate models, it has not been determined whether neurons can recover arbors after direct dendrite damage.

Whether dendrites can regenerate has been addressed most directly in invertebrate model systems. *Drosophila* dendritic arborization (da) neurons have well-defined dendrites [18] that share many cellular features with mammalian dendrites [19, 20]. These cells are a good model for studying injury responses because they are optically accessible in living animals, and their axons regenerate using conserved machinery, including dual leucine zipper kinase (DLK), the axon injury sensor [21]. Using laser microsurgery, individual dendrites can be severed from da neurons [22–24]. Initially it was suggested that only da neurons with large dendrite arbors (class IV) could regenerate dendrites, while neurons with smaller arbors (class I) could not [22, 24]. Subsequent studies showed that removal of one or all dendrites in both classes of da neurons triggered regeneration [21, 25]. Both larval and adult da neurons can regenerate dendrites [21, 26], although the capacity is reduced as animals age [26]. Dendrite repair after laser injury has also been demonstrated in *Caenorhabditis elegans*, although the process involves plasma membrane fusion to reattach the cutoff piece [27] and so may be different from regeneration in other animals.

Very little is known about the intrinsic molecular machinery that promotes dendrite regeneration. Akt was identified as a general growth-promoting factor for axons and dendrites after injury [24]. However, several other pathways that are required for axon regeneration have been shown not to play a role in dendrite regeneration. Importantly, DLK, a conserved regulator of axon injury signaling [28–31], and downstream proteins c-Jun N-terminal kinase (JNK) and fos are not required for dendrite regeneration [21]. Indeed, signaling through this pathway is not initiated by dendrite injury [21]. Thus, dendrite injury is likely to be sensed by different machinery than axon injury. Even after early signaling, the two processes diverge: spastin is required for the growth stage of axon regeneration to coordinate endoplasmic reticulum (ER) and microtubule concentration at the regenerating axon tip, but dendrite regeneration is not sensitive to spastin reduction and does not involve ER concentration at growing tips [32, 33]. Thus, at both the initiating and growth stages, axon and dendrite regeneration diverge in molecular requirements.

In order to identify the machinery specifically required for dendrite regeneration, we focused on receptor tyrosine kinases (RTKs) because these proteins trigger other cellular growth responses. Using the best-established model for dendrite regeneration, we conducted an RNA interference (RNAi) screen of RTKs. We found that one RTK, RTK-like orphan receptor (Ror), was required for dendrite regeneration in class I and class IV *Drosophila* da neurons, but not for axon regeneration. Ror is an intriguing RTK that was for many years an orphan but finally emerged as a Wnt receptor/coreceptor that can influence a variety of Wnt signaling pathways in vertebrates [34–36]. Ror proteins have a cysteine-rich domain that can bind Wnt ligands, and they can also interact with the 7-transmembrane classic Wnt receptors, frizzleds (fzs) [34–36]. A duplication in the vertebrate lineage has given rise to two Ror genes, and both have been linked to nervous system development [34–36]. In mice, Ror proteins are required for many actions of Wnt5a through regulation of Dishevelled2 (Dvl2) phosphorylation [37]. Outputs of the pathway beyond Dvl2 have been elusive. Ror2 localizes to growing neurites and mature dendrites of hippocampal neurons [38–40] and has been linked to spine formation [38] and synaptic transmission [40]. The *C*. *elegans* Ror protein CAM-1 can also act postsynaptically and functions together with fz upstream of Dishevelled (Dsh) [41]. *Drosophila* has one Ror protein and a second similar protein, neurospecific receptor kinase (Nrk), that is sometimes considered part of the Ror family and alternatively more closely related to the muscle-specific kinase (MuSK) family [42]. Both are found specifically in the nervous system [43–45]. While Nrk is required for normal eye development [46], homozygous null *Ror* mutant animals are viable and have no reported phenotypes [45]. *Drosophila* Ror does, however, bind Wnt ligands and an fz receptor [45]. In this study, we link *Drosophila* Ror to dendrite regeneration and show that a downstream target of the Ror–Dsh pathway is microtubule nucleation in dendrites.

## Results

### A screen to identify RTKs that control dendrite regeneration

Regeneration of the dendrite arbor of the class IV dorsal da neuron ddaC has been described in several studies [21, 25]. After complete dendrite removal using laser microsurgery, degeneration of severed dendrites is largely complete by 4 h, and a new arbor has extended in a radius of several hundred microns by 48 h [21]. By 96 h after injury, the arbor has covered its normal territory in the body wall [21]. To identify proteins involved in dendrite regeneration, we knocked down candidates using cell-type–specific RNAi and assayed arbor size 24 h after dendrite removal. We compared several different measures of regeneration to identify one that would balance efficiency and information content. Maximum diameter of the dendrite arbor was a reasonable proxy for total arbor length (S1 Fig) and was therefore chosen to measure regenerative growth.

**Fig 1:**
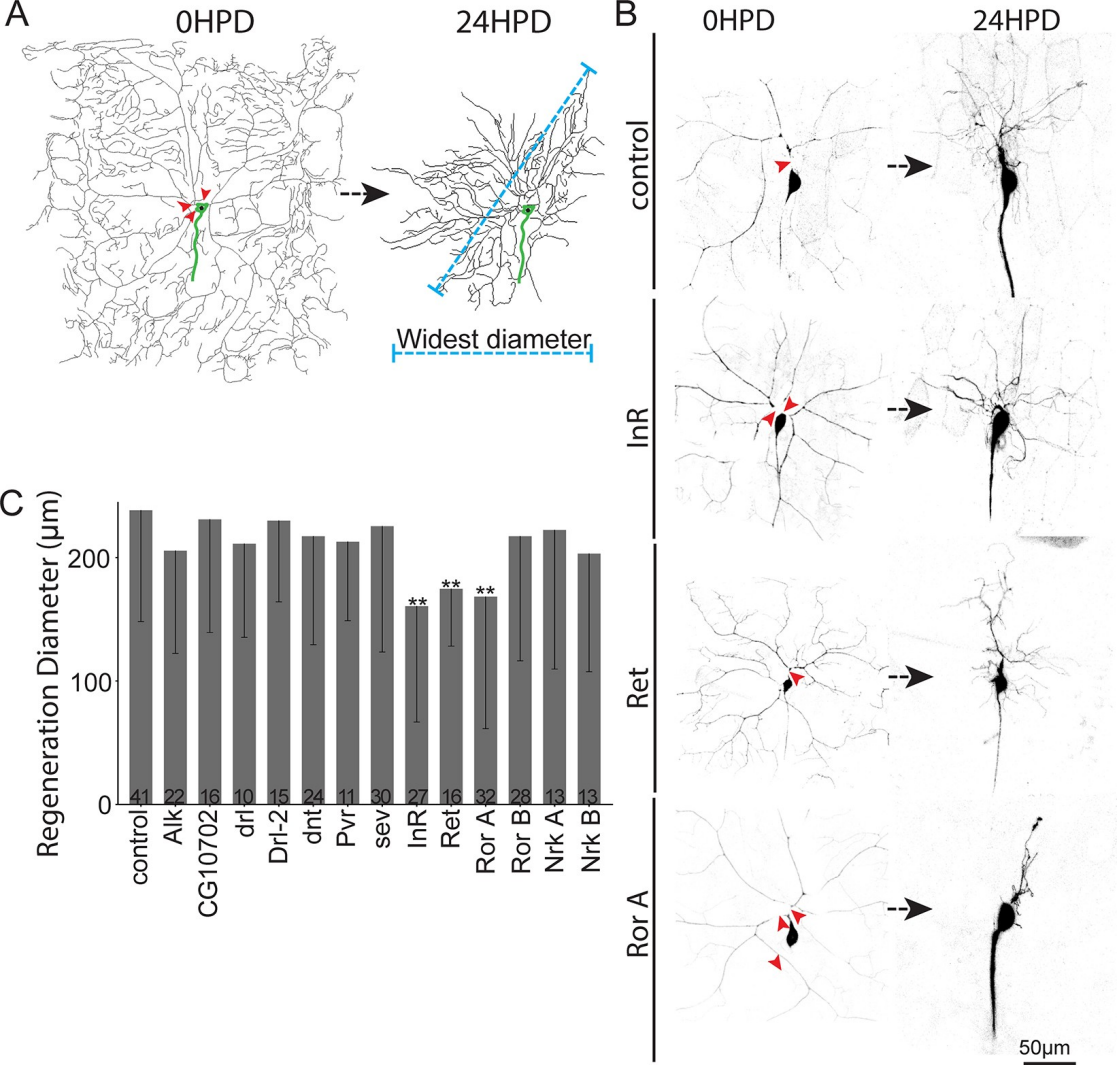
Identification of RTKs involved in dendrite regeneration. (A) Full dendrite arbor trace of a ddaC neuron before (left) and 24 h after (right) dendrite removal with a pulsed UV laser (red arrowheads). Regeneration was quantified 24 HPD using the widest diameter of the regenerating arbor (blue dotted line). The cell body and beginning of the axon are indicated in green. (B) Representative ddaC neurons immediately after dendrites were severed (left) and 24 HPD (right). Cells were labeled using the tester line ppk-Gal4, UAS-Dicer2;UAS-mCD8-GFP crossed to lines expressing the respective individual RNAi hairpins. The control targets the maternal γTub37C transcript not present in somatic tissues like neurons. (C) Quantification of dendrite regeneration. Bars show mean regeneration, error bars represent standard deviation. Sample size (within bar, bottom) represents number of cells analyzed, with one cell per animal. *P < 0.05, **P < 0.01, ***P < 0.001 with Mann–Whitney U test to compare mean rank. Quantitation is contained in S1 Data. Alk, anaplastic lymphoma kinase; da, dendritic arborization; ddaC, dorsal da neuron C; dnt, doughnut on 2; drl, derailed; GFP, green fluorescent protein; HPD, hours postdendrotomy; InR, insulin-like receptor; Nrk, neurospecific receptor kinase; ppk, pickpocket; Pvr, PDGF- and VEGF-receptor related; Ret, Ret oncogene; RNAi, RNA interference; Ror, RTK-like orphan receptor; RTK, receptor tyrosine kinase; sev, sevenless; UAS, upstream activating sequence; γTub, γTubulin.

RTKs were selected as a candidate gene set based on their involvement in growth pathways. In *Drosophila*, there are 20 genes in the RTK family [47]. To select the best candidates from within this group, we performed transcriptome analysis on small pools [48, 49] of ddaC neurons microdissected from larvae. Ten RTKs were expressed above our threshold for selection (S2 Fig). Anaplastic lymphoma kinase (Alk) was added to this set because it has previously been associated with growth control under nutrient stress [50]. Transcripts encoding these kinases were targeted for knockdown in class IV neurons using the Gal4-upstream activating sequence (UAS) system to express hairpin RNAs. Dicer2 was expressed to enhance neuronal RNAi [51], and mCD8-green fluorescent protein (GFP) was used as a cell-shape marker. Three-day–old larvae were mounted for laser surgery, and a pulsed UV laser was used to sever all dendrites from a single ddaC neuron per animal (Fig 1). Twenty-four h after injury, control neurons regenerated arbors that averaged over 200 μm in diameter (Fig 1). Arbor size was significantly reduced in neurons expressing RNA hairpins targeting the insulin-like receptor (InR), Ret oncogene (Ret), and Ror (Fig 1). We concluded that these 3 RTKs could play a role in dendrite regeneration.

In order to identify genes likely to be generally required for dendrite regeneration, we performed a secondary screen. When the large comb-shaped dendrite of the class I ddaE neuron is removed, the cell responds by adding branches to remaining dendrites so that several days later, the preinjury number of branch points is recapitulated [21]. At the time injury is performed, uninjured ddaE neurons no longer add branches [21], so the branch point addition after injury is a change in cellular behavior that likely requires sensing the injury and reinitiating a growth program. The amount of growth required is, however, substantially less than that in the ddaC assay, so we reasoned that ddaE regeneration might be less sensitive to genes required broadly for large-scale outgrowth.

**Fig 2:**
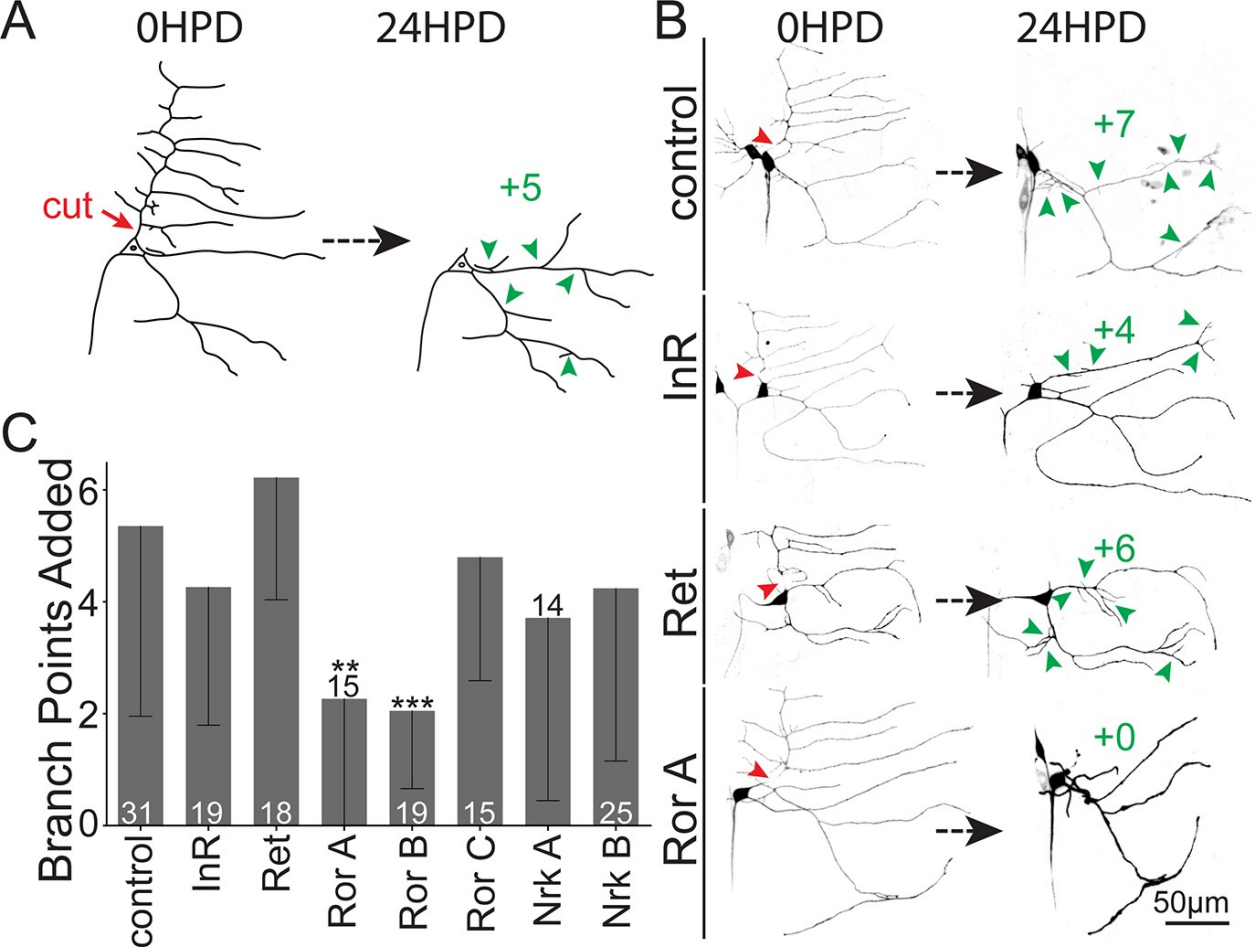
Secondary screen of RTKs required for dendrite regeneration. (A) Assay for ddaE dendrite regeneration. The dorsal comb dendrite was severed (red arrowhead) at time 0. The cell was imaged 24 h later, and new branches (green arrowheads) were counted. (B) Representative images of neurons expressing Dicer2, mCD8-GFP, and RNAi hairpins under control of the 221-Gal4 driver are shown. (C) Quantification of dendrite regeneration in neurons expressing different RNAi hairpins. Regeneration is represented by the number of new branch points 24 HPD; error bars are standard deviation; sample size (within bar, bottom) is number of animals from which one neuron was injured. *P < 0.05, **P < 0.01, ***P < 0.001 with Mann–Whitney U test to compare mean rank. Quantitation is contained in S1 Data. da, dendritic arborization; ddaE, dorsal da E; GFP, green fluorescent protein; HPD, hours postdendrotomy; InR, insulin-like receptor; Nrk, neurospecific receptor kinase; Ret, Ret oncogene; RNAi, RNA interference; Ror, RTK-like orphan receptor; RTK, receptor tyrosine kinase.

**Fig 3:**
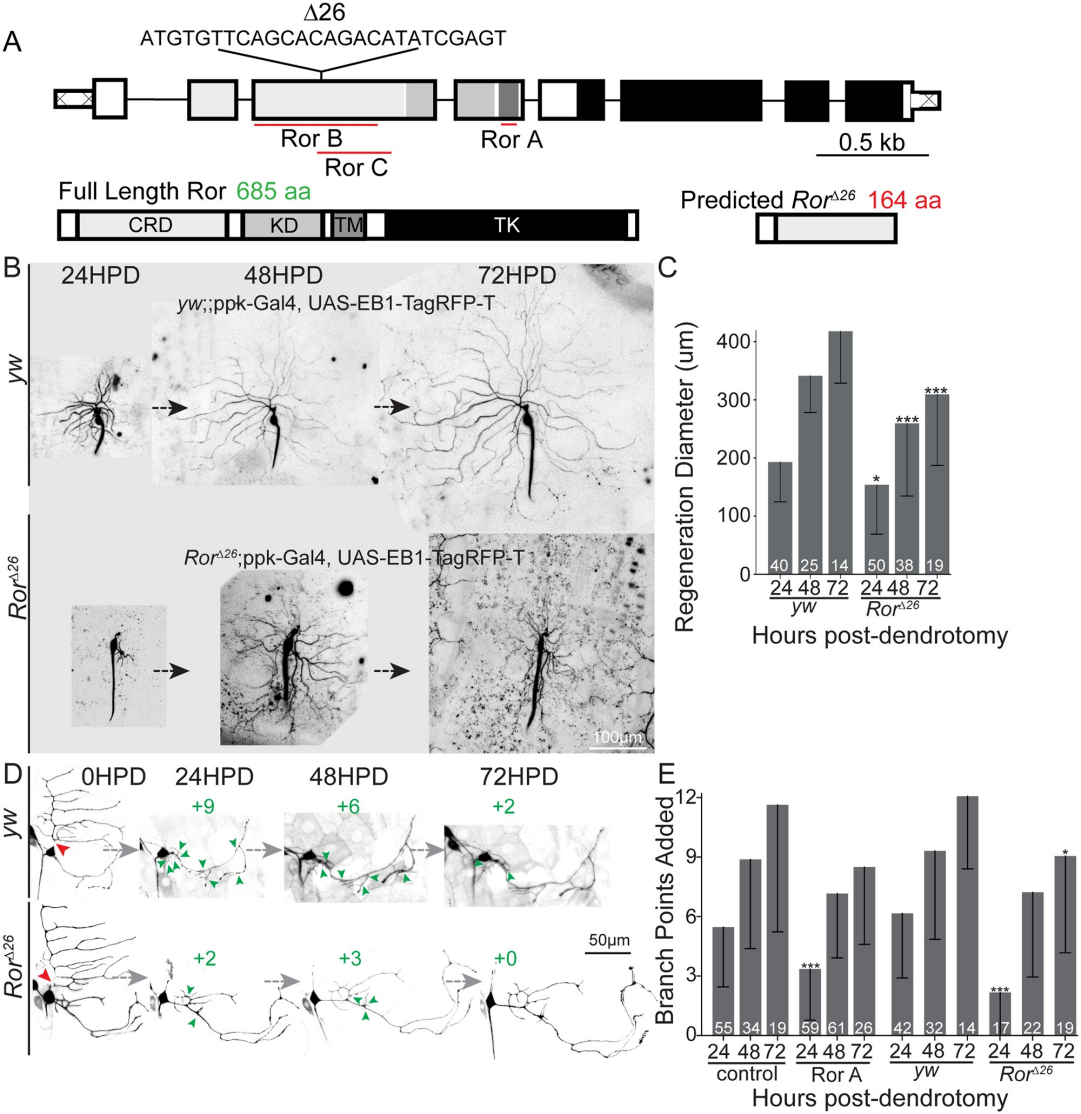
Ror is required for normal dendrite regeneration in ddaC and ddaE neurons. (A) Diagram of the *Ror* gene and protein with 26-bp deletion resulting in null *Ror*^Δ26^ mutant. The target regions of RNAi hairpins used in this work are mapped onto the gene. Domains in the protein include fz-like CRD, KD, TM, and TK. (B) Representative ddaC dendrite regeneration over the course of 72 HPD. The mutant line was *Ror*^*Δ26*^*/*^*Δ26*^;ppk-Gal4, UAS-EB1-TagRFP-T crossed to *Ror*^*Δ26*^*/*^*Δ26*^. Control flies used were *yw*;ppk-Gal4, UAS-EB1-TagRFP-T crossed to *yw*. Each neuron was imaged every 24 h to quantify regeneration over time in *yw* (control) or *Ror*^*Δ26*^ null mutants. Quantitation is shown in (C). (D) Representative dendrite regeneration over time in ddaE neurons. Dorsal comb dendrites were severed (red arrowhead), and new branchpoints (green arrowheads) were counted at 24 h intervals. Mutant phenotype: *Ror*^*Δ26*^*/*^*Δ26*^;221-Gal4, UAS-mCD8-GFP crossed to *Ror*^*Δ26*^*/*^*Δ26*^. Control flies used were *yw*;221-Gal4, UAS-mCD8-GFP crossed to *yw*, and quantitation is shown in (E). Graphs show mean regeneration diameter, with error bars showing standard deviation (C) or added branch points (E). Sample size (within bar, bottom) represents individual neurons from each animal. *P < 0.05, **P < 0.01, ***P < 0.001 with Mann–Whitney U test to compare mean rank. Quantitation is contained in S1 Data. CRD, cysteine-rich domain; da, dendritic arborization; ddaC/E, dorsal da C/E; EB1, end binding protein 1; fz, frizzled; GFP, green fluorescent protein; HPD, hours postdendrotomy; KD, kringle domain; ppk, pickpocket; RFP, red fluorescent protein; RNAi, RNA interference; Ror, RTK-like orphan receptor; RTK, receptor tyrosine kinase; TK, tyrosine kinase domain; TM, transmembrane domain; *yw*, *yellow*, *white*.

To test for the roles of Ret, Ror, InR, and Nrk (a Ror RTK family member) in ddaE dendrite regeneration, we crossed transgenic lines containing UAS-driven RNA hairpins to a tester line (UAS-Dicer2;221-Gal4, UAS-mCD8-GFP) and severed the comb dendrite with a pulsed UV laser. New branches added to the remaining dendrites were counted 24 h later (Fig 2A). Control neurons added an average of over 5 branches in this time period, while those expressing two independent Ror hairpins (target regions in Ror gene are shown in Fig 3A) added an average of about two branch points (Fig 2B and 2C). No reduction was seen for a third Ror RNAi line or those targeting InR, Ret, or Nrk (Fig 2B and 2C). We concluded that Ror was the best candidate for further analysis as a regulator of dendrite regeneration.

### Ror is required for dendrite regeneration in class I and IV neurons

To confirm a role for Ror in dendrite regeneration, we wished to use mutant as well as RNAi tools and to assay the effect of Ror reduction at longer times after dendrite injury. The *Ror*^*Δ26*^ mutant has a 26-bp deletion that is predicted to result in a frameshift and stop codon within 10 codons of the deletion (Fig 3A). Like flies homozygous for the recently described *Ror*^*4*^ null mutation [45], homozygous *Ror*^*Δ26*^ flies are viable, healthy, and fertile and show no signs of a planar cell polarity phenotype based on analysis of wing hair orientation (S3 Fig). Standard genetic techniques were used to introduce class I and IV neuronal markers into *Ror*^*Δ26*^ mutant animals, and dendrite regeneration was assayed. *Ror* mutant animals recapitulated the RNAi phenotype of reduced regeneration in both ddaC and ddaE neurons at 24 h after injury (Fig 3B and 3E). In addition, in both *Ror* mutant animals and animals in which Ror was specifically knocked down in neurons, regeneration remained lower than in control animals 3 days after injury (Fig 3B and 3E). Thus, Ror functions cell autonomously and is required for normal levels of dendrite regeneration.

### Dendrite development is normal in Ror mutant animals

**Fig 4:**
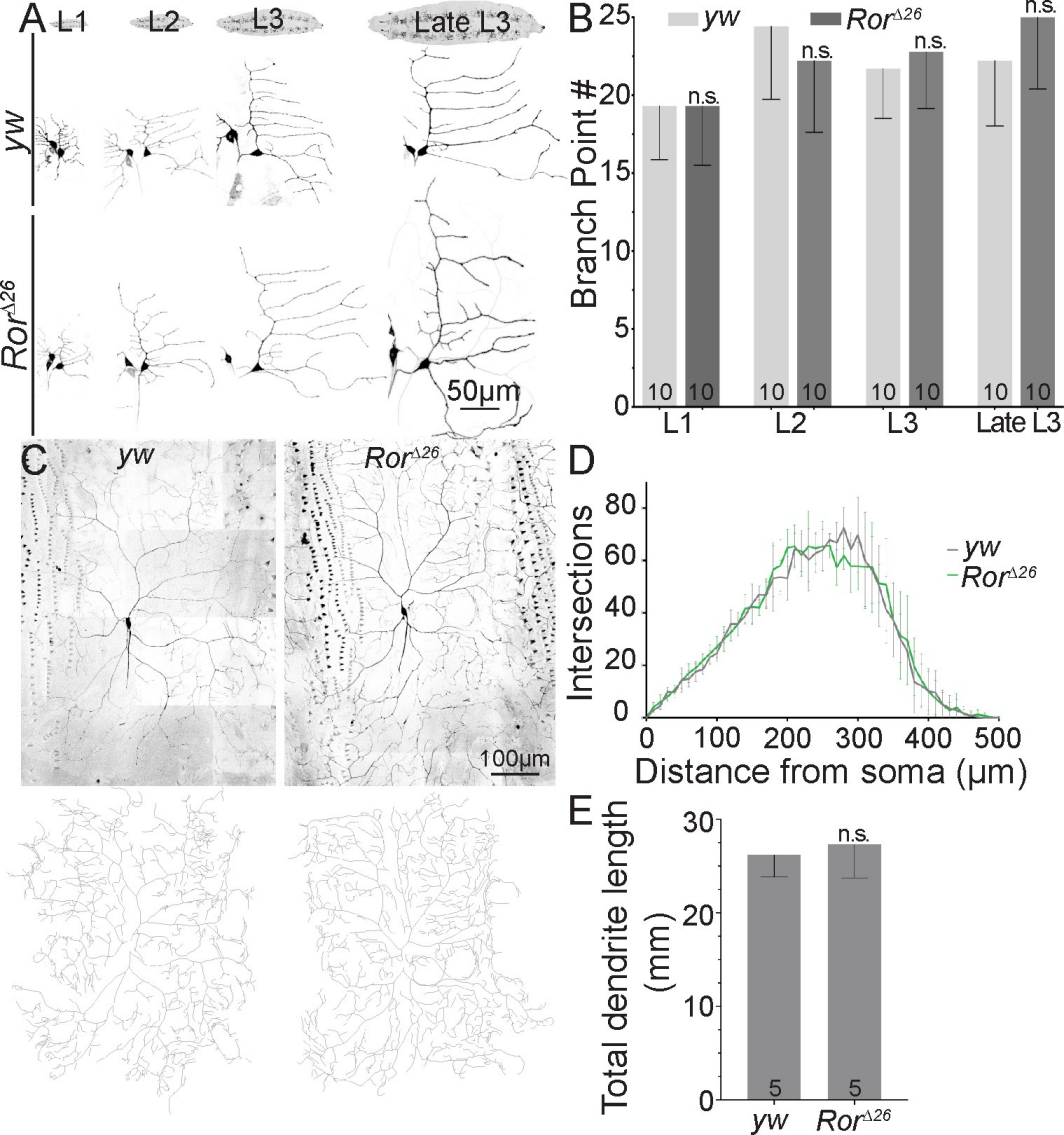
Ror is not required for dendrite development. (A) Images of ddaE neurons throughout larval development are shown from *yw* (control) or *Ror*^*Δ26*^ mutant animals. Cells expressed mCD8-GFP under control of 221-Gal4. (B) Quantification of branch point number during ddaE development. Values are the mean of 10 animals per group. Error bars show standard deviation. (C) Representative images (top) and traces (bottom) of ddaC neurons from 3-day–old larvae. A Sholl analysis is shown in (D) and total dendrite length in (E). For the Sholl analysis, no difference was observed between nonlinear regression curves fitted as a fifth-order polynomial. Total dendrite length was unaffected in *Ror* mutants compared to controls using unpaired *t* test. Numbers on the bars indicate number of animals used. Quantitation is contained in S1 Data. da, dendritic arborization; ddaC/E, dorsal da C/E; GFP, green fluorescent protein; n.s., not significant; Ror, RTK-like orphan receptor; RTK, receptor tyrosine kinase; *yw*, *yellow*, *white*.

Although neurons were not obviously misshapen when they were severed for dendrite regeneration assays, we wished to more rigorously determine whether Ror loss affected developmental dendrite outgrowth. We therefore compared branch point number in ddaE at different developmental stages in wild-type and *Ror* mutant animals. Even early in larval development, when defects in initiation of outgrowth should be most obvious, no difference was seen (Fig 4A and 4B). ddaC neurons continue to add branches throughout larval development, so they were only assayed at one time point. No defects in dendrite complexity or length were observed in *Ror* mutant animals (Fig 4C and 4D). This lack of developmental phenotype is consistent with the morphologically normal central nervous system in *Ror*^*4*^ mutant animals [45]. We conclude that Ror loss does not affect normal dendrite development.

### Ror reduction does not affect axon regeneration

**Fig 5:**
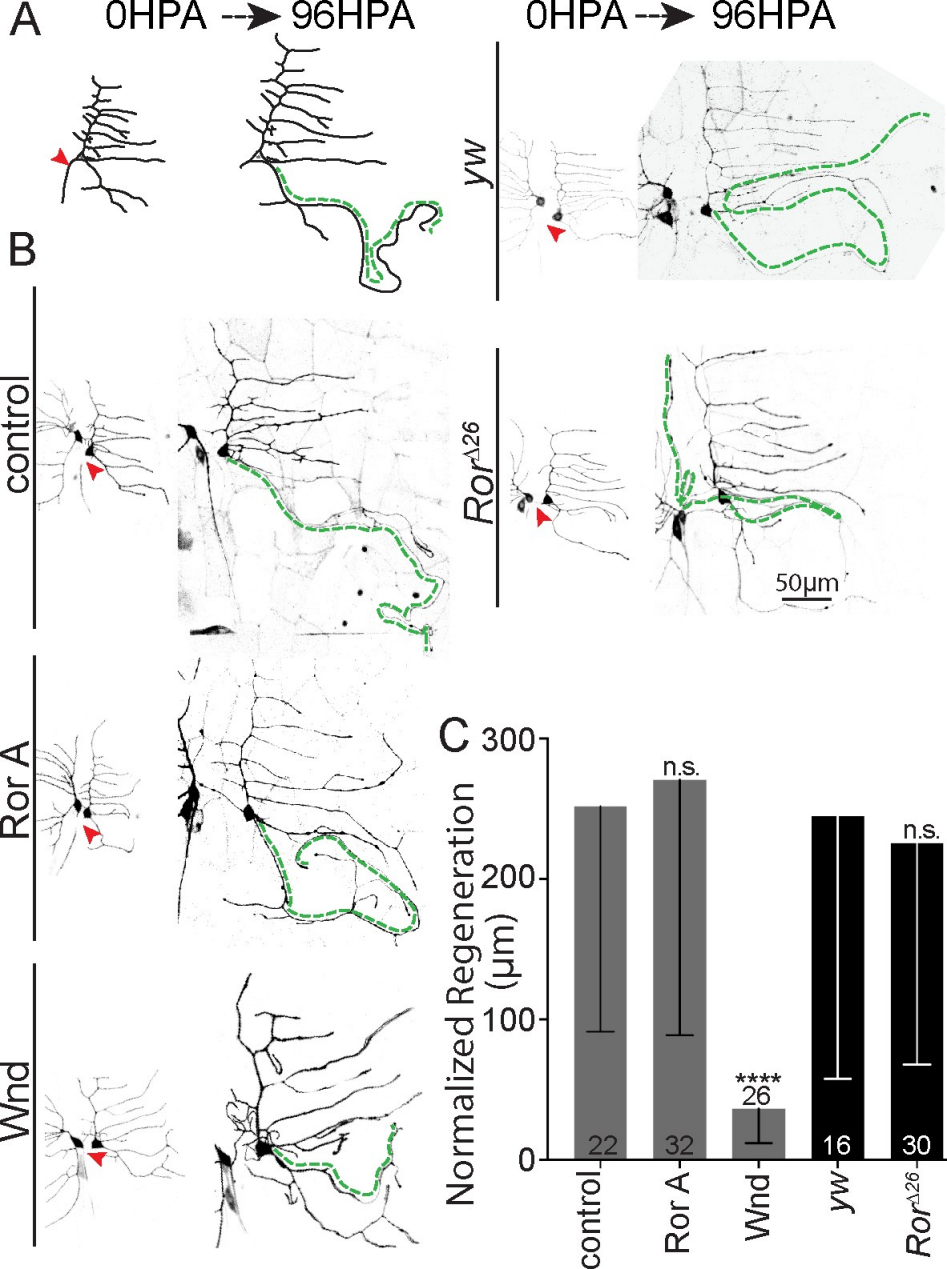
Axon regeneration is normal in *Ror* mutants. (A) Diagram of ddaE axon regeneration, which occurs via conversion of a dendrite to a growing axon after proximal axon injury. Growth of the converted dendrite from the tip is measured 96 HPA. (B) Representative images of ddaE neurons at 0 h and 96 HPA in control and Ror knockdown neurons labeled with 221-Gal4,UAS-mCD8-GFP and expressing dicer2 and hairpin RNAs (for lower images). The green dotted line indicates converted dendrite. (C) Quantification of axonal regeneration normalized to arbor size. Only Wnd/DLK RNAi showed near-total abrogation of regeneration phenotype (*P* < 0.001). Mann–Whitney U test, *P < 0.05, **P < 0.01, ***P < 0.001. Bars show mean, and error bars are standard deviation; sample size represents individual animals/neurons. Quantitation is contained in S1 Data. da, dendritic arborization; ddaE, dorsal da E; DLK, dual leucine zipper kinase; GFP, green fluorescent protein; HPA, hours postaxotomy; RNAi, RNA interference; Ror, RTK-like orphan receptor; RTK, receptor tyrosine kinase; UAS, upstream activating sequence; wnd, wallenda; *yw*, *yellow*, *white*.

To determine whether Ror is specifically required for dendrite regeneration or more broadly for regrowth after injury, we assayed axon regeneration in ddaE neurons. In both vertebrates and invertebrates, after axons are severed close to the cell body, the stump is not competent for regeneration, and instead, a dendrite is converted into a regenerating axon (Fig 5A) [23, 52]. For da neurons, this means that in most cases, the regenerating axon remains near the surface of the animal and can be easily measured [32, 33]. Occasionally the converted axon encounters the nerve and tracks along it, indicating that it responds to pathfinding cues, as expected for a regenerating axon [53]. DLK (wallenda [wnd] in flies) is required for axon regeneration in these neurons [21] and was used as a positive control (Fig 5B and 5C). Unlike wnd RNAi, which eliminated axon regeneration, Ror RNAi did not affect the amount of axon outgrowth (Fig 5B and 5C). Furthermore, no defect in axon regeneration was observed in *Ror* mutant animals (Fig 5B and 5C). Thus, Ror reduction seems specifically to affect dendrite regeneration and not dendrite development or axon regeneration.

### Wnt receptors and downstream scaffolding proteins are required for normal dendrite regeneration

**Fig 6:**
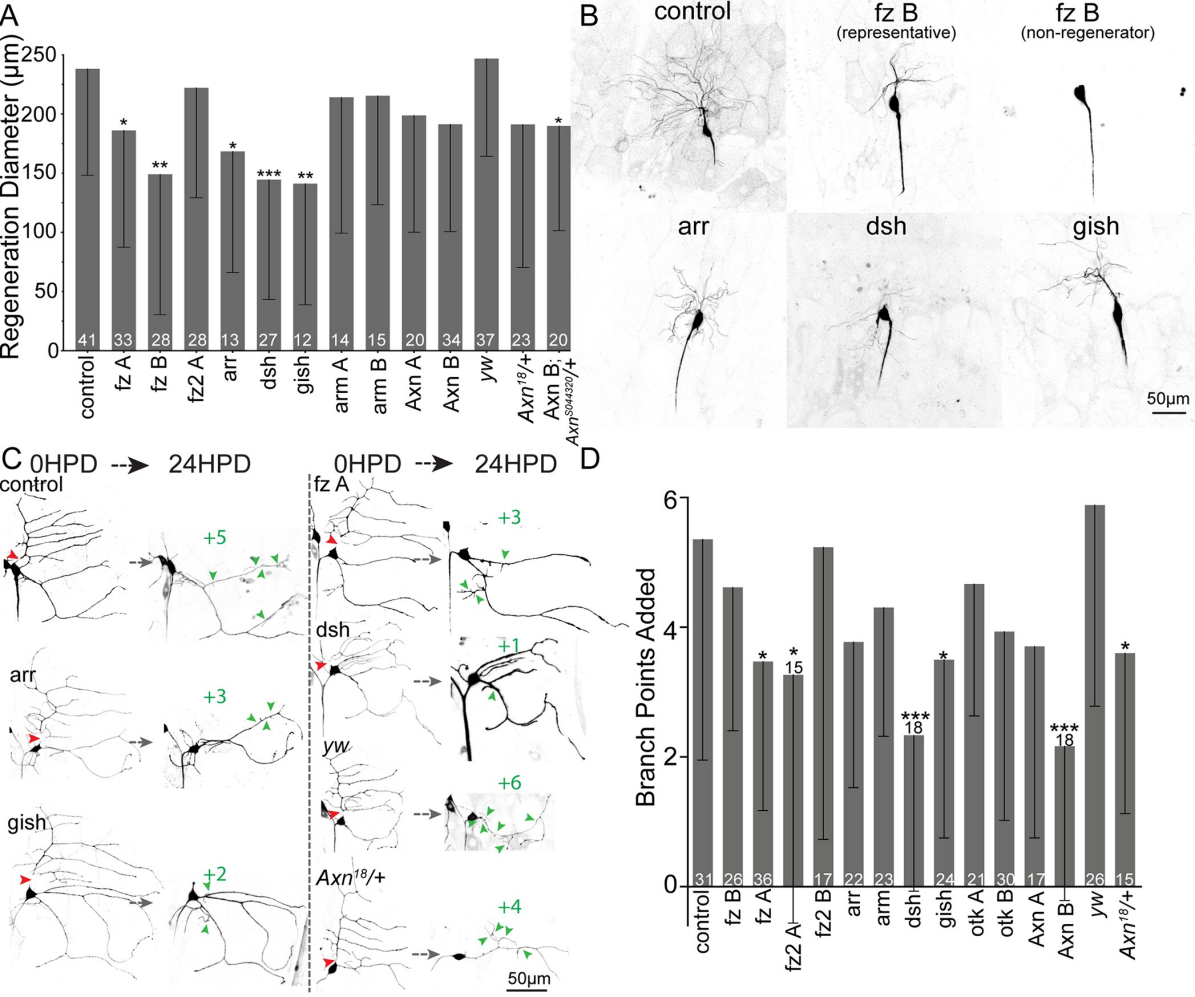
Wnt signaling proteins contribute to dendrite regeneration. (A) Quantification of ddaC dendrite regeneration diameter 24 HPD after RNAi knockdown of Wnt signaling proteins. (B) Example images of ddaC neurons 24 HPD are shown. (C) Representative images of ddaE regeneration following knockdown of Wnt signaling proteins. (D) Quantification of ddaE branchpoints added 24 HPD. Mann–Whitney U test, *P < 0.05, **P < 0.01, ***P < 0.001. Error bars show standard deviation; sample size (within bar, bottom) represents individual animals/neurons. Quantitation is contained in S1 Data. arm, armadillo; arr, arrow; Axn, Axin; da, dendritic arborization; ddaC/E, dorsal da C/E; dsh, dishevelled; fz, frizzled; gish, gilgamesh; HPD, hours postdendrotomy; otk, off-track; RNAi, RNA interference; *yw*, *yellow*, *white*.

In *C*. *elegans* and vertebrates, Ror has been linked to Wnt signaling cascades [34–36], and in flies, Ror has been shown to bind to several Wnt ligands and to frizzled2 (fz2) [45]. We therefore hypothesized that Wnt signaling proteins may function with Ror in dendrite regeneration. To test this hypothesis, we used RNAi to reduce Wnt signaling proteins in ddaC and ddaE neurons and assayed dendrite regeneration. Many, but not all, RNAi hairpins targeting Wnt signaling proteins reduced dendrite regeneration (Fig 6). Regeneration in ddaE neurons seemed slightly more sensitive to Wnt signaling protein knockdown than regeneration in ddaC neurons. In ddaE, at least one RNAi hairpin targeting both fzs, the Wnt coreceptor arrow (arr), downstream scaffolds Axin and dsh, and gish (gilgamesh) (casein kinase 1γ [CK1γ]) resulted in fewer added branches 24 h after injury (Fig 6). We confirmed a role for Axin using animals heterozygous for a null allele (Fig 6C and 6D) or one copy of a null allele paired with Axin RNAi (Fig 6A). These results suggest that Ror could be acting to regulate dendrite regeneration as part of a Wnt signaling pathway.

### Ror is required to localize Wnt signaling scaffold proteins to dendrites, and this requirement is enhanced during dendrite regeneration

**Fig 7:**
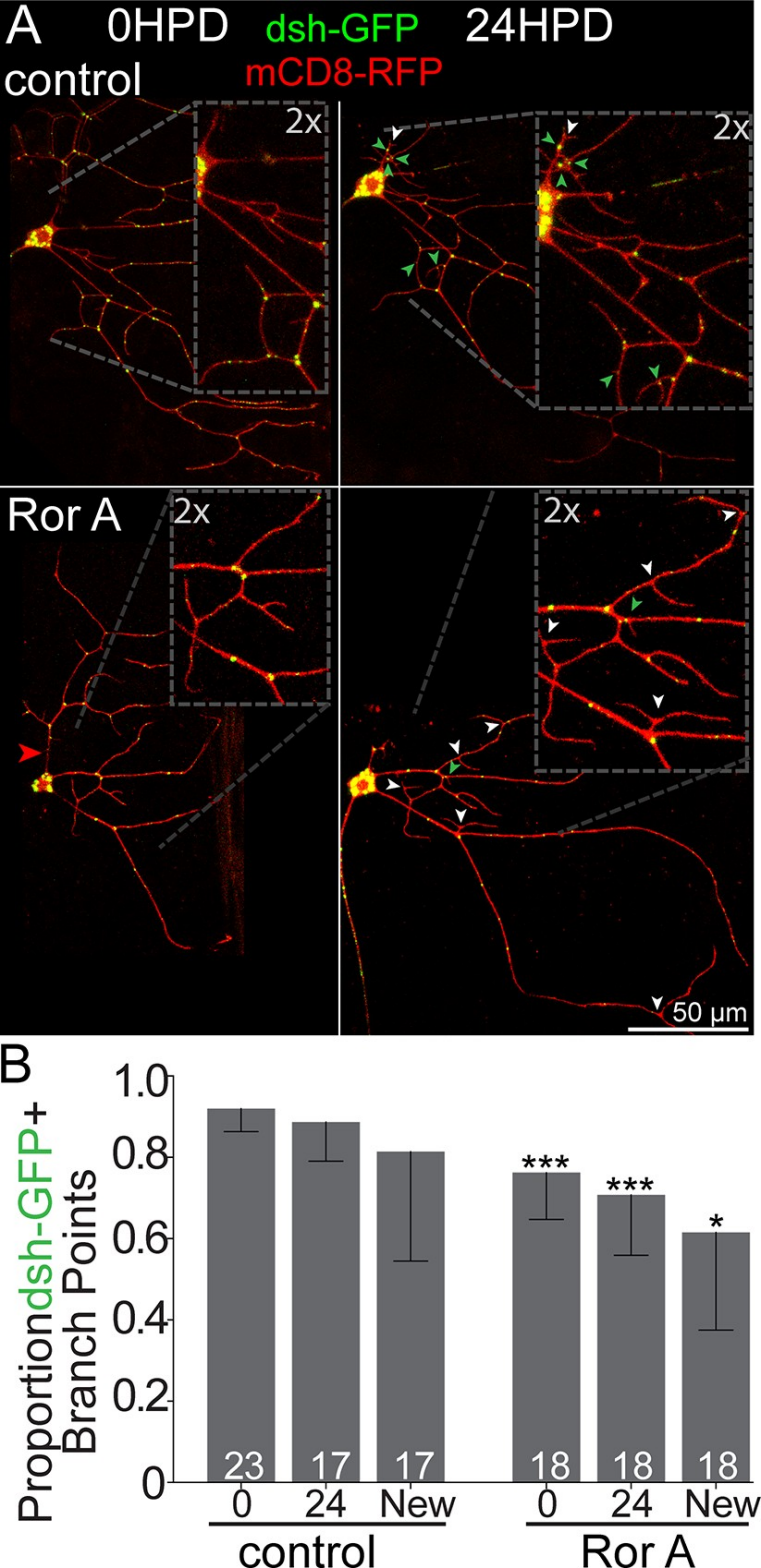
dsh localization at baseline and during dendrite regeneration. (A) UAS-dsh-GFP expressed under control of 221-Gal4 with UAS-mCD8-mRFPas a cell-shape marker at baseline (left) and 24 HPD (right). Red arrowheads denote cut sites; green arrowheads show new branch points positive for dsh-GFP, while white arrowheads show GFP-negative new branch points. (B) Quantification of dsh-GFP+ branchpoints at baseline and 24 HPD in control and Ror RNAi. For 24 HPD, the total number of branch points with dsh-GFP is shown in the 24 column, and newly added branch points are also shown separately. Mann–Whitney U test, *P < 0.05, **P < 0.01, ***P < 0.001. Bars show mean; error bars are standard deviation; sample size represents individual animals/neurons. Quantitation is contained in S1 Data. dsh, dishevelled; GFP, green fluorescent protein; HPD, hours postdendrotomy; RNAi, RNA interference; Ror, RTK-like orphan receptor; RTK, receptor tyrosine kinase; UAS, upstream activating sequence.

We have recently found that Wnt signaling proteins act at dendrite branch points to control microtubule nucleation [54]. Both Axin and dsh concentrate in puncta at branch points in ddaE neurons and are in turn required to concentrate the core microtubule nucleation protein γTubulin at branch points [54]. To determine whether Ror acts upstream of dsh in dendrites, we knocked down Ror in ddaE neurons expressing dsh-GFP [55] together with a membrane marker and Dicer2. In control neurons, dsh-GFP localizes to tight puncta at dendrite branch points (Fig 7A). In Ror knockdown neurons fewer, branch points contained dsh puncta (Fig 7A and 7B). Axin-GFP [55] is also concentrated at dendrite branch points and was similarly affected by Ror knockdown (S4 Fig). These results suggest Ror acts upstream of dsh and Axin in dendrites in uninjured neurons, although the reduction in localization was partial.

To test whether dendrite regeneration would impose a stronger requirement for Ror on localization of Wnt signaling scaffolds, we assayed dsh-GFP localization at dendrite branch points after injury. In control neurons, dsh-GFP was efficiently added to new branch points after removing the comb dendrite from ddaE (Fig 7A, green arrowheads, and 7B). In contrast, fewer new branch points contained dsh puncta in Ror knockdown neurons (Fig 7A, white arrowheads, and 7B). Overall, these data suggest that Ror is important for localizing Wnt signaling proteins to dendrites, and this function may be more stringently required after injury.

We also tested whether we detect Ror in dendrites before and after injury. Using a Ror-GFP transgene expressed using its own promoter [45], we could detect expression of Ror in da neurons, including dendrites, although the signal was very weak (S5A Fig). As well as a diffuse signal enriched in neurons, some brighter spots were present. To determine whether they might be endosomes, we paired Ror-GFP with mCherry-Rab5, a red Rab5 expressed from its own regulatory regions [56]. While we did observe some colocalization (S5B Fig), at such low fluorescence signals, we could not rule out that this was due to fluorescence bleedthrough or autofluorescent spots with broad excitation and emission spectra. After dendrite injury, it was not possible to unambiguously detect Ror-GFP signal in dendrites, perhaps because of bleaching from the laser used to injure the neurons and the thinness of new dendrites. We also found that the Ror-GFP transgene could not rescue the mutant phenotype in regeneration assays (S5C Fig), so we did not pursue additional localization studies with it.

### Ror controls dendrite regeneration by positioning nucleation sites at new branch points

**Fig 8:**
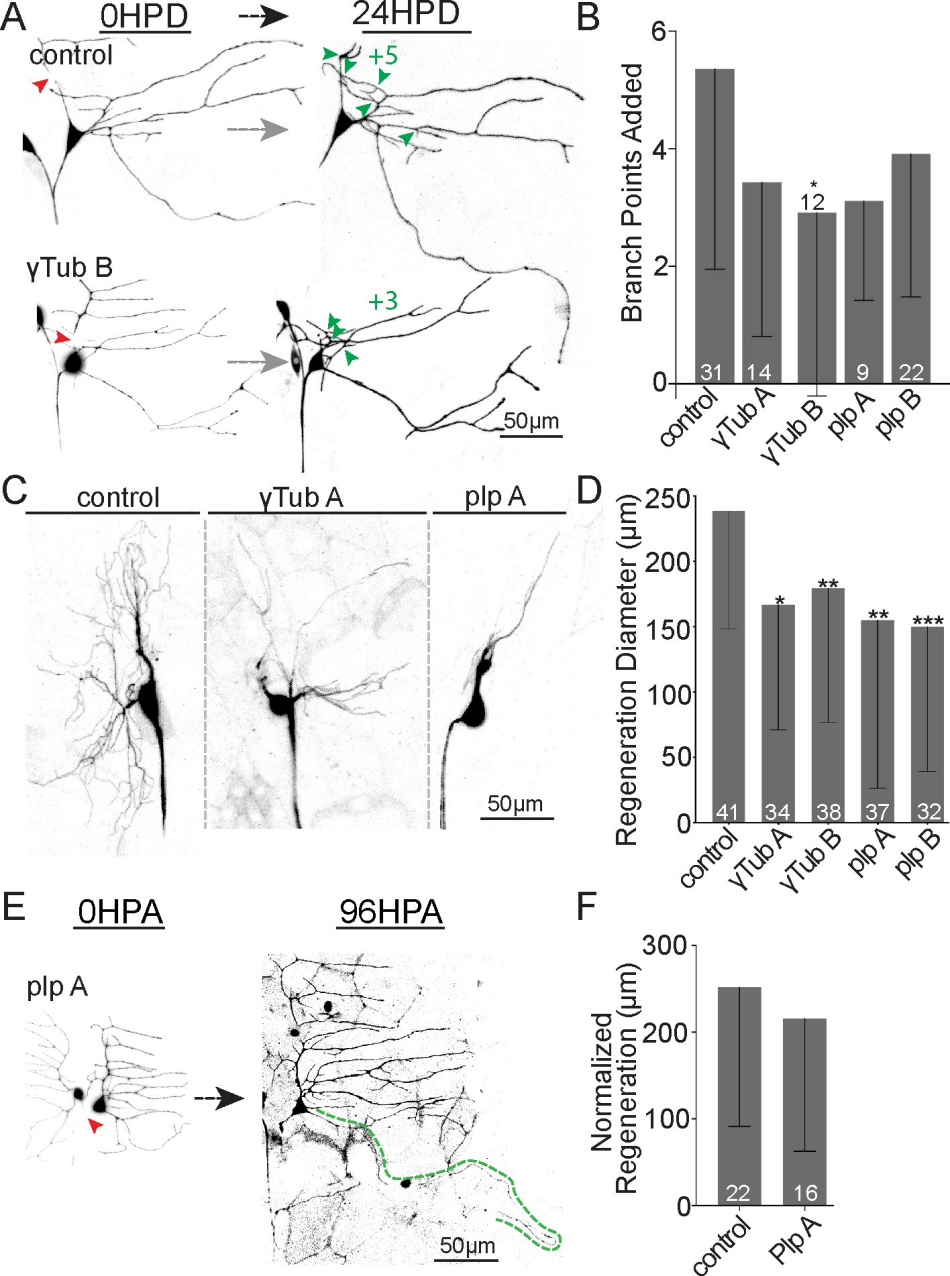
Microtubule nucleation is required for normal dendrite regeneration. (A) Representative images of ddaE dendrite regeneration in control, ɣTub, and plp RNAi neurons. (B) Number of branch points added 24 HPD is shown. (C) Representative ddaC arbors 24 HPD expressing RNAi hairpins targeting plp and γTub. (D) Quantitation of ddaC dendrite regeneration diameter 24 HPD is shown. (E) Example images of ddaE axon regeneration in Plp RNAi neurons are shown. (F) Mean normalized regeneration length (μm) is shown in the graph. Note: axon regeneration control data set is repeated (F) from Fig 5 for comparison. Error bars are standard deviation; sample size (within bar, bottom) represents individual animals/neurons. Mann–Whitney U test, *P < 0.05, **P < 0.01, ***P <0.001. Quantitation is contained in S1 Data. da, dendritic arborization; ddaC/E, dorsal da C/E; HPA, hours postaxotomy; HPD, hours postdendrotomy; plp, pericentrin-like protein; RNAi, RNA interference; γTub, γTubulin.

We have identified local microtubule nucleation as a key output of Wnt signaling proteins in dendrites [54]. We therefore hypothesized that Ror might act through Wnt signaling proteins to help nucleate microtubules in dendrites and that this function might be important for normal dendrite regeneration. To test this hypothesis, we first examined whether dendrite regeneration is sensitive to partial reduction of the core nucleation protein γTubulin. We have previously shown axon regeneration is not sensitive to a 50% reduction in γTubulin using heterozygous mutants or RNAi [14]. In contrast, dendrite regeneration in ddaC and ddaE was impaired by γTubulin RNAi (Fig 8). To gain additional support for sensitivity of dendrite regeneration to microtubule nucleation, we knocked down pericentrin-like protein (plp). plp has previously been linked to dendritic microtubule nucleation [57], and is required for γTubulin localization to dendrite branch points [54]. plp RNAi also dampened dendrite regeneration (Fig 8) and, like Ror and γTubulin RNAi, did not affect axon regeneration (Fig 8). These results are consistent with a specific requirement for microtubule nucleation in supporting dendrite regeneration.

Microtubule nucleation has also been reported to affect dendrite arbor shape in large ddaC neurons [57], although not in smaller ddaE neurons [58]. If all microtubule nucleation was controlled by Ror, we might expect to see a similar reduction in dendrite arbor complexity in Ror mutant animals, but this was not the case (Fig 4C and 4D). We therefore examined the impact of Wnt signaling pathway members that reduced dendrite regeneration on normal arbor development. Like Ror, reduction of Fz or Dsh did not have an effect on arbor complexity or total arbor length (S6 Fig); the results for Axin, which is a more downstream component of the pathway [54] were ambiguous, with perhaps a slight reduction in the arbor (S6 Fig). Thus, Ror and the other upstream Wnt signaling proteins seem to play a more specific function in dendrite regeneration than γTubulin and possibly Axin. One potential explanation for this is that Ror and upstream Wnt signaling proteins are only required for local nucleation in dendrites, while γTubulin and perhaps Axin are globally required for nucleation.

**Fig 9:**
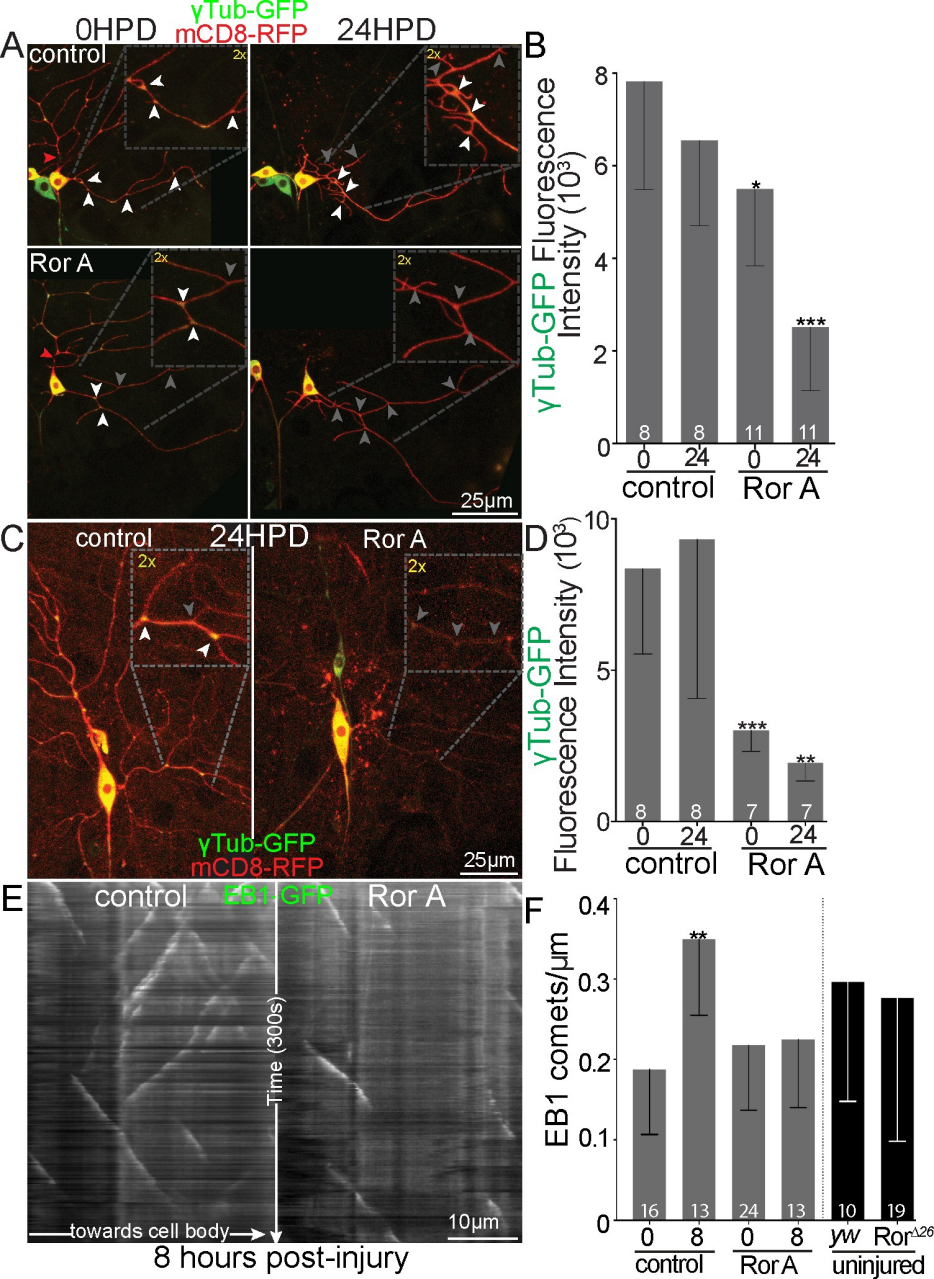
Localization of γTub-GFP and a functional assay for nucleation in control and Ror RNAi neurons. (A) Representative images of ddaE neurons expressing UAS-mCD8-RFP, UAS-Dicer2, and UAS-ɣTub-GFP under 221-Gal4 control. Neurons are shown before dendrite removal and 24 HPD. The cut sites are shown with red arrowheads. Areas of high-signal γTub-GFP at branch points are indicated with white arrowheads; low signal is indicated with gray arrowheads. (B) Quantitation of γTub-GFP fluorescence at branch points was calculated as (BP − nBP) = (fluorescence); insets show 2× magnification of indicated branch points. Sample size (within bar, bottom) represents individual neurons/animals. Linear regression, *P < 0.05, **P < 0.01, ***P < 0.001. (C) Representative images of ddaC neurons expressing UAS-γTub-GFP and UAS-mCD8-RFP under ppk-Gal4 control are shown. White arrowheads indicate branch points with normal γTub-GFP signal, and gray arrowheads indicate low signal. (D) Quantification of γTub-GFP at branch points was calculated as in B. (E) Kymographs of EB1-GFP in dendrites of ddaE neurons are shown in E 8 h after axon injury. Kymographs show white EB1 comet tracking over 300 s. (F) Microtubule dynamics was quantified as EB1-GFP comet number in the comb dendrite of ddaE dendrite during 300 s live imaging (widefield, Zeiss; 63× Oil PlanApo 1× zoom). *yw* was used as control for *Ror*^*Δ26*^ animals. *P <0.05, **P < 0.01, ***P < 0.001; Mann–Whitney U test. Numbers on bars of all graphs are numbers of cells analyzed. Quantitation is contained in S1 Data. da, dendritic arborization; ddaC/E, dorsal da C/E; EB1, end binding protein 1; GFP, green fluorescent protein; HPD, hours postdendrotomy; ppk, pickpocket; RFP, red fluorescent protein; RNAi, RNA interference; Ror, RTK-like orphan receptor; RTK, receptor tyrosine kinase; UAS, upstream activating sequence; *yw*, *yellow*, *white*; γTub, γTubulin.

To further pin down whether nucleation downstream of Ror might be important locally in dendrites, we assayed dendritic localization of γTubulin in control and Ror RNAi neurons before and during dendrite regeneration. In uninjured neurons, Ror RNAi partially reduced γTubulin at dendrite branch points (Fig 9), and during dendrite regeneration, very little γTubulin was seen at branch points (Fig 9). This assay makes use of an overexpressed but functional γTubulin-GFP [58]. In order to test whether the reduction at branch points might be due to an overall change in γTubulin levels rather than a change in dendritic localization, we used a γTubulin tagged at the endogenous locus with super-folder GFP (sfGFP) [59]. Fluorescence levels of γTubulin-sfGFP were measured in the cell bodies of control and Ror RNAi ddaE neurons that were colabeled with spectrally well-separated iBlueberry (S7 Fig). There was not a significant difference in cell body levels, so we favor a model in which Ror and other Wnt signaling proteins act in dendrites to localize nucleation sites to branch points. To further test this idea, we made use of a functional assay for dendritic nucleation. Axon injury triggers up-regulation of nucleation in dendrites, and one readout of this is an increase in the number of microtubule plus ends visualized by the plus-end–binding protein end binding protein 1 (EB1) [14]. In control neurons, the number of microtubule plus ends per unit length of dendrite increases about 2-fold 8 h after axon injury (Fig 9). This increase is completely suppressed by Ror RNAi (Fig 9). Note that in uninjured neurons, no difference in plus-end number was seen in Ror RNAi or mutant neurons, consistent with function being specifically important under stress scenarios requiring nucleation. Overall, we conclude that dendrite regeneration is sensitive to reduced levels of nucleation proteins and that Ror controls localization of these proteins to dendrites.

## Discussion

Using a candidate screening approach, we identified Ror as a regulator of dendrite regrowth after injury in two different *Drosophila* neurons. Axon regeneration and developmental dendrite outgrowth were unaffected in *Ror* mutant animals. This injury-specific phenotype is consistent with expression of Ror in the nervous system [43, 45] without detectable defects in nervous system architecture in mutants [45]. In *C*. *elegans* and mammals, Ror has been associated with some subtypes of Wnt signaling [34–36], and in flies, it has been shown to bind Wnt ligands and fz2 [45]. Based on this link between Ror and Wnt signaling, we tested Wnt signaling proteins for a role in dendrite regeneration. The subset that affected regenerative growth included those involved broadly in Wnt signaling like fzs, gish (CK1γ), and dsh and those involved more specifically in canonical Wnt signaling like arr, low-density lipoprotein related-receptor protein 5/6 (LRP5/6), and Axin but notably did not include armadillo (arm; β-catenin), the output of canonical Wnt signaling. Serendipitously, this subset matched the proteins we identified in a screen for factors required to position microtubule nucleation sites in dendrites [54]. So, although the outputs of Ror-mediated Wnt signaling have typically been elusive, we were able to show that Ror acts upstream of dendritic microtubule nucleation, and this likely mediates its effect on dendrite regeneration. Overall, we propose a model in which Ror acts together with other Wnt receptors to localize the scaffolding proteins dsh and Axin to dendrites, and these, in turn, promote microtubule nucleation throughout dendrite arbors. Because microtubules are structural elements and tracks for transport, generation of microtubules throughout dendrites is likely required for rapid regrowth after injury.

Microtubule nucleation also occurs locally in axons [58, 60, 61] and in uninjured dendrites [57, 58, 62], so why are axon regeneration and dendrite development normal in *Ror* mutants? For axon regeneration, one possibility is that Wnt signaling proteins do not act upstream of nucleation in this compartment. However, axon regeneration is also more resistant to partial loss of γTubulin than dendrite regeneration is, suggesting that axon regeneration is generally less sensitive to nucleation levels than dendrite regeneration rather than just resistant to Ror loss. Alternatively, the relatively high stability of axonal microtubules relative to dendritic ones [63, 64] could account for the difference in sensitivity to reduced nucleation. Axonal microtubules tend to be longer and turn over less than dendritic ones, meaning that the demands on nucleation may be lower in axons. If this is the case, a different explanation is needed for the resilience of dendrite development to Ror loss. One possibility is that Ror only functions to position nucleation sites during dendrite regeneration, but not during development. However, this does not seem to be the case because γTubulin-GFP branch point localization was lower in uninjured dendrites when Ror was knocked down (Fig 8). Instead, we favor the idea that Ror-mediated nucleation acts in parallel to other pathways that are sufficient for microtubule generation in uninjured neurons. For example, microtubule severing followed by Patronin-mediated microtubule minus-end growth could maintain microtubule number under normal conditions. Patronin is a microtubule minus-end–binding protein [65, 66] that facilitates persistent growth of minus ends into dendrites during development and regeneration [67]. In *C*. *elegans*, Patronin has been shown to act in parallel to nucleation [68]. If a new microtubule were released from a nucleation site by a severing protein and recognized by Patronin, the nucleation site could act catalytically, and very few would be required. Only under extreme conditions, such as those imposed by regrowth of dendrites after injury, would local nucleation become essential.

While the specific deficit of dendrite regeneration in *Ror* mutants could be due to increased demands on nucleation that surpass a phenotypic threshold only after dendrite injury, the fact that Ror is a signaling receptor raises the intriguing possibility that it could also respond to injury signals. The closest phylogenetic neighbors to the Ror family of RTKs are the tropomyosin receptor kinases (Trks) [69], which have been lost in evolution in flies and worms [70, 71]. One major function of Trk receptors is to couple neuronal survival to target innervation. Target tissues secrete neurotrophins that bind Trks on neurons to generate signaling endosomes that are transported to the cell body to promote survival [70]. The involvement of Ror in dendrite regeneration suggests that it could also link a neuronal survival/adaptation response to the state of surrounding cells. Ror function has been tightly linked to Wnt binding [34–36, 42, 72], so it is likely that a Wnt is also involved in this context. Wnt5a-Ror signaling can work in an autocrine loop [73], but because neuronally expressed Wntless RNAi does not affect γTubulin localization [54], we think it more likely that a ligand is secreted from a surrounding cell. It is therefore possible that surrounding cells influence dendrite regeneration through Ror-controlled microtubule nucleation.

## Materials and methods

### *Drosophila* stocks

Many *Drosophila* stocks used in this study were acquired from Bloomington Drosophila Stock Center (NIH P40OD018537) and the Vienna Drosophila Resource Center. Refer to S1 Table for information regarding all fly lines used.

Tester lines used in this study include (UAS-Dcr2, UAS-mCD8-RFP;221-Gal4, UAS-dsh-GFP), (UAS-Dcr2, UAS-mCD8-RFP;221-Gal4, UAS-γtub-GFP), (UAS-Dcr2;221-Gal4, UAS-EB1-GFP), (UAS-Dcr2, UAS-mCD8-RFP;221-Gal4, UAS-Axin-GFP), (UAS-Dcr2;ppk-Gal4, UAS-mCD8-GFP), (Ror^Δ26^;ppk-Gal4, UAS-mCD8-GFP), (Ror^Δ26^;ppk-Gal4, UAS-EB1-TagRFPT), (Ror^Δ26^;221-Gal4, mCD8-GFP), and (UAS-Dcr2;221-Gal4, UAS-mCD8-GFP). Virgin female flies from tester lines were combined with males from RNAi or mutant stocks in fly cross vials to obtain desired genotype. For a complete list of fly stocks, including all RNAi lines used, please see S1 Table. Food caps containing standard fly food media with proprionic acid and methylparaben (tegosept) were collected every 24 h. Caps with embryos/larvae were reared for 72 h at 25 °C. Fly food media (ingredients for 10 L) included 45 g agar, 259 g sucrose, 517 g dextrose, 155 g yeast, 858 g cornmeal, 40 mL 10% tegosept in ethanol, and 60 mL proprionic acid.

### Identification of the *Ror*^*Δ26*^ allele

*Ror*^*Δ26*^ was identified during the sequencing of the *bsk*^*2*^ chromosome [74], which also contains a lethal mutation in the neighboring *bsk* gene [75]. The two mutations were separated by screening 1.5 × 10^5^ meiotic recombinants, and the isolated *Dror*^*Δ26*^ mutant chromosomes were found to be homozygous viable and viable in combination with a deficiency uncovering *Ror*, *Df(2L)170B*.

### Class I dendrite regeneration assay

Larvae were aged at 25 °C for 72 h. Individual larvae were cleaned in PBS, mounted on a microscope slide with a dried agarose pad, and transiently immobilized with a coverslip (#40, 0.1 mm; VWR International, Radnor, PA, USA) and tape. Larvae were imaged using Zeiss microscopes (LSM 700, LSM 800, AxioImager M2; Carl Zeiss, Oberkochen, Germany) and injured with a pulsed UV laser (Andor, Oxford Instruments, Abingdon, UK) using a 63× 1.4 NA objective. Postinjury, larvae were returned to fresh fly food at 20 °C for 24 h unless otherwise indicated. Individual larvae were remounted at the specified times after injury and imaged with a 40× 1.4 NA objective on a Zeiss LSM800 confocal microscope controlled with Zen Blue software. Regeneration was scored as (total branch points at 24 h postinjury) − (branch points at time of injury). For longer time points (Fig 3), postinjury branch point number was subtracted from the total branch points at each time to generate branch points added after injury.

### Class IV dendrite regeneration assay

L3 larvae were maintained at 25 °C until injury. For ddaC neurons, primary dendrites were severed via pulsed UV laser proximal to the first branch point. After injury, the larva was placed in fresh fly food and kept at 25 °C for 24 h unless otherwise indicated. The larva was then cleaned in PBS, and the dendritic field was acquired as a maximum intensity projection of z-stack confocal images (Zeiss LSM800, Zen Blue software). Regeneration was quantified at point of maximum regeneration (widest diameter) of the dendrite arbor.

### Axon regeneration assay

Class I ddaE neurons were visualized via expression of UAS-mCD8-GFP under control of the 221-Gal4 [76]. Two-day–old larvae were mounted on microscope slides on top of a dried agarose pad and secured beneath a coverslip (#40, 0.1 mm; VWR International) using tape. Proximal axotomy (5–20 μm from soma) was performed similarly to dendrotomy. Immediately postinjury, the larva was returned to fresh fly food at 20 °C for 96 h. Axon regeneration was quantified as the amount of new axon growth normalized to the increase in larval size as measured by change in length of an un-converted dendrite according to the following formula:

Regeneration_tip growth_ = Length_new axon_ − (Length_0h_ × (Length_96h control dendrite_/Length_0h control dendrite_)). Regenerating axons can be identified by microtubule polarity and tip growth [23, 32–33]. In cases in which regeneration is impaired, the nearest dendrite is measured.

### Protein localization and fluorescence quantification

Images for protein localization experiments before or after injury were acquired on a Zeiss LSM 800 confocal microscope. Maximum intensity projections of z-stacks were used for all localization analyses. UAS-dsh-GFP was quantified on a binary basis with branch points that had a single dsh-GFP puncta or none. For the nonpunctate markers UAS-γTub-GFP or UAS-Axin-GFP, the area between branch points (nBP) and branch point (BP) values were collected and measured as described in Weiner and colleagues [54]. y-axis values shown on the graphs indicate the BP − nBP fluorescent value.

### Quantification of class IV dendrite arbor

Quantification of total dendrite length was performed using the Fiji distribution of ImageJ [77] simple neurite tracer plugin [78] and adding the length of all paths corresponding to dendrites. Sholl analysis was performed using the Sholl analysis plugin [79] on traced skeletons of dendrites in 10-μm steps with no normalization for volume.

### Statistical analysis and data visualization

GraphPad Prism (V7 and 8) software was used for statistical analysis and visualization. Figures were made using Adobe Illustrator. Statistical significance was reported as *P < 0.05, **P < 0.01, ***P < 0.001, with error bars representing standard deviation. Statistical analyses were developed in collaboration with the Penn State Statistical Consulting Center. For dendrite regeneration and protein localization experiments, individual comparisons were made using Mann–Whitney U tests to compare mean ranks with no assumption of how the data were distributed. For dendrite arbor characterization, an unpaired *t* test was used to compare total dendrite length and Sholl analysis data. Axon regeneration data were similarly compared using unpaired *t* tests with the assumption that the data were similarly distributed. For initial characterization and design of regeneration screening assays, regeneration data were analyzed using the Kolmogorov–Smirnov test, which compares cumulative distributions and can detect differences in data distributions. See figure legends for the specific statistical test used in each case.

## Supporting information

S1 Fig Dendrite regeneration can be accurately screened using a proxy metric for rearborization. (A) Representative image showing class IV dendrite regeneration screening assay denoting cut sites (red arrows) and regenerated arbor 24 HPD. Full characterization via arbor tracing (B) is laborious. We designed a proxy metric for expedited screening by comparing regeneration diameter at the widest point of the arbor to total arbor length (C). The values tracked well for distributions seen in our assays but might overestimate small values and underestimate robust regenerators, but the metric functions well as an expedient screening tool for additional investigation. Quantitation is contained in S1 Data. HPD, hours postdendrotomy.
(TIF)

S2 Fig Expression levels of the 20 RTKs in *Drosophila* in microdissected ddaC neurons. Transcriptomes were generated from microdissected ddaC soma after RNA isolation, cDNA conversion, and library preparation. RNA sequencing of 5′ skewed reads were mapped to transcript libraries and represent mRNA counts within neurons. Bars represent average transcript counts (log scale) and error bars SEM of 4 independent samples. A cutoff value was established (red segmented line) for screening of RTKs that might be important in neuronal processes. Quantitation is contained in S1 Data. da, dendritic arborization; ddaC, dorsal da C; RTK, receptor tyrosine kinase.
(TIF)

S3 Fig Ror is not involved in PCP signaling. (A) Diagrams of normal (left) and perturbed (right) hair morphology on adult *Drosophila* wings. Wing hair organization is downstream of PCP signaling and is disrupted in strong fz loss-of-function mutants (B). Wing hair organization was assayed in *Ror* mutants and compared to *fz* mutants. 10–15 wings from adult male and female flies were imaged under high magnification (40×–100×, variable) on a Zeiss AxioZoom injection microscope under brightfield illumination. Images were acquired as z-stacks using CellSens software and processed in ImageJ and Adobe Photoshop by Focus Stacking for representative images to improve clarity. Blinded analyses could discern aberrant wing hair phenotypes only in strong *fz* loss-of-function animals (*Fz*^*F31/P21*^). *Ror* mutants showed no discernable PCP phenotype. Quantitation is contained in S1 Data. fz, frizzled; PCP, planar cell polarity; Ror, RTK-like orphan receptor; RTK, receptor tyrosine kinase.
(TIF)

S4 Fig Ror knockdown alters Axin localization in dendrites. (A) Example images of ddaE neurons expressing UAS-Axin-GFP and UAS-mCD8-RFP. White arrows indicate BPs with normal Axin signal and gray arrows those with low signal. Insets show 2× magnification of indicated BPs. (B) Quantification of Axin-GFP fluorescence intensity was normalized as follows: BP − nBP = normalized FI. Multiple linear regression analysis; *P < 0.05, **P < 0.01, ***P < 0.001. Bars represent mean normalized FI ± SD; sample size (within bar, bottom) represents individual neurons/animals. Quantitation is contained in S1 Data. da, dendritic arborization; ddaE, dorsal da E; BP, branch point; FI, fluorescence intensity; GFP, green fluorescent protein; nBP, non-BP; RFP, red fluorescent protein; Ror, RTK-like orphan receptor; RTK, receptor tyrosine kinase; UAS, upstream activating sequence.
(TIF)

S5 Fig Ror localization in peripheral neurons. Transgenic Ror-GFP under control of the endogenous Ror promoter was visualized in larva expressing no other fluorophores (A) as homozygous Ror-GFP in a wild-type background. Weak Ror fluorescence can be discerned in soma, axons, and proximal dendrites of the dorsal arborization cluster. Animals with a single copy of the Ror-GFP allele show similar expression patterns as homozygous animals (B), and coexpression with transgenic Rab5-mCherry under its endogenous promoter shows potential colocalization in a subset of Rab5-labeled endosomes. However, we cannot rule out bleedthrough due to residual excitation of mCherry by 488-nm light. Images were acquired on an LSM800 inverted confocal microscope (63× oil plan-apo, 3× zoom) while anesthetized. (C) Dendrite regeneration in ddaE neurons homozygous for *RorΔ26* and expressing transgenic Ror-GFP show impaired regeneration similar to homozygous mutants, suggesting transgenic Ror-GFP may be nonfunctional. Control and Ror mutant data are repeated from Fig 3 for comparison. Rescue experiment: *Ror*^*Δ26*^*/*^*Δ26*^;221-mCD8-GFP crossed to *Ror*^*Δ26*^_*/*_^*Δ26*^;Ror-p-Ror-GFP. Graphs show added branch points over 24, 48, and 72 h. Sample size (within bar, bottom) represents individual neurons from each animal. *P < 0.05, **P < 0.01, ***P P < 0.001 with Mann–Whitney U test to compare mean rank. Quantitation is contained in S1 Data. da, dendritic arborization; ddaE, dorsal da E; GFP, green fluorescent protein; Ror, RTK-like orphan receptor; RTK, receptor tyrosine kinase.
(TIF)

S6 Fig Class IV dendrite arbor shape in different genetic backgrounds. Class IV ddaC neurons expressing 1407-Gal4, ppk-Gal4, ppk-CD4-tdGFP;elav-Gal4, UAS-Dicer2, and respective UAS-RNAi hairpins were imaged in third-instar larva while anesthetized to create maximum intensity projections (A). Full arbor traces were obtained using the SNT plugin for ImageJ. Sholl analyses were acquired using the Sholl Analysis feature of SNT at 10-μm intervals (B). Total dendrite length (C) was compared using a Mann–Whitney U test to compare ranks. *P < 0.05, **P < 0.01, ***P < 0.001. Numbers on the bars are numbers of cells analyzed, and error bars are standard deviation. Quantitation is contained in S1 Data. da, dendritic arborization; ddaC, dorsal da C; GFP, green fluorescent protein; ppk, pickpocket; RNAi, RNA interference; SNT, Simple Neurite Tracer; tdGFP, tandem dimer GFP; UAS, upstream activating sequence.
(TIF)

S7 Fig Endogenous γTub levels in control and Ror RNAi neurons. Class I neurons expressing a far-red fluorophore (UAS-iBlueberry, controlled by 221-Gal4) and endogenously tagged γTub-sfGFP and either control RNAi (A, top) or Ror RNAi 62868 (A, bottom). Total γTub levels were quantified as GFP fluorescence in a single optical slice from z-stacks acquired of the ddaE soma. Expression of endogenous γTub is too low to be reliably quantified in dendrites. Mean GFP FI was acquired in ImageJ, and levels (B) did not appreciably differ in Ror RNAi animals versus control. *P < 0.05, **P < 0.01, ***P < 0.001; Mann–Whitney U test. Numbers on the bars are numbers of cells analyzed, and error bars show standard deviation. Quantitation is contained in S1 Data. da, dendritic arborization; ddaE, dorsal da E; FI, fluorescence intensity; GFP, green fluorescent protein; RNAi, RNA interference; Ror, RTK-like orphan receptor; RTK, receptor tyrosine kinase; sfGFP, super-folder GFP; UAS, upstream activating sequence; γTub, γTubulin.
(TIF)

S1 Data Data used to generate the figures in this work are provided in supporting information file S1 Data. Quantitation is sorted by figure (workbook tabs) and corresponding legend in the A1 cell. Additional information is supplied above each individual data set for clarity. Legends correspond to figure panels such that S1 Data sheet “Figure 1” contains all data used to make that figure.
(XLSX)

S1 Table A complete list of all *Drosophila* stocks used is provided in the table, together with their source. (XLSX)

S1 Supplemental Methods A file with methods used to generate Supporting Information Figures. (DOCX)

## Abbreviations

Alk: anaplastic lymphoma kinase
arm: armadillo
arr: arrow
CK1γ: casein kinase 1γ
da: dendritic arborization
ddaC/E: dorsal da neuron C/E
DLK: dual leucine zipper kinase
dnt: doughnut on 2
drl: derailed
dsh: dishevelled
Dvl2: Dishevelled2
EB1: end binding protein 1
ER: endoplasmic reticulum
fz: frizzled
GFP: green fluorescent protein
gish: gilgamesh
HPA: hours postaxotomy
HPD: hours postdendrotomy
InR: insulin-like receptor
JNK: c-Jun N-terminal kinase
LRP5/6: low-density lipoprotein related-receptor protein 5/6
MuSK: muscle-specific kinase
Nrk: neurospecific receptor kinase
otk: off-track
plp: pericentrin-like protein
ppk: pickpocket
Pvr: PDGF- and VEGF-receptor related
Ret: Ret oncogene
RNAi: RNA interference
Ror: RTK-like orphan receptor
RTK: receptor tyrosine kinase
sev: sevenless
sfGFP: super-folder GFP
TBI: traumatic brain injury
TRiP: transgenic RNAi project
Trk: tropomyosin receptor kinase
UAS: upstream activating sequence
wnd: wallenda
yw: *yellow*, *white*
