# Supplementary figures and images for: The receptor tyrosine kinase Ror is required for dendrite regeneration in Drosophila neurons
Source: PLoS Biol. 2020 Mar 12;18(3):e3000657. doi: 10.1371/journal.pbio.3000657 (PMC7067388; doi:10.1371/journal.pbio.3000657)

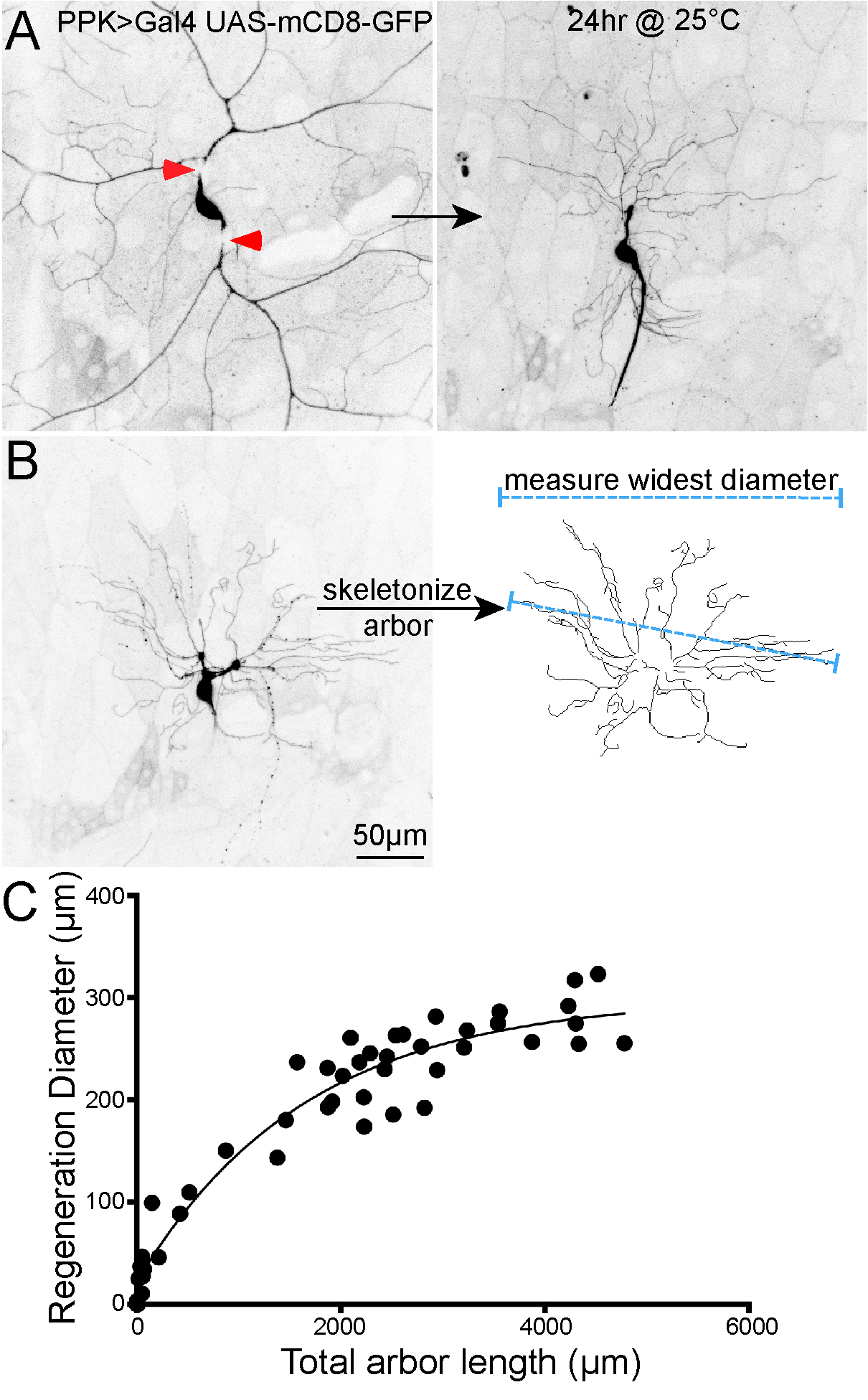

Supplement: S1 Fig — (A) Representative image showing class IV dendrite regeneration screening assay denoting cut sites (red arrows) and regenerated arbor 24 HPD. Full characterization via arbor tracing (B) is laborious. We designed a proxy metric for expedited screening by comparing regeneration diameter at the widest point of the arbor to total arbor length (C). The values tracked well for distributions seen in our assays but might overestimate small values and underestimate robust regenerators, but the metric functions well as an expedient screening tool for additional investigation. Quantitation is contained in S1 Data. HPD, hours postdendrotomy. (TIF) [file pbio.3000657.s001.tif]

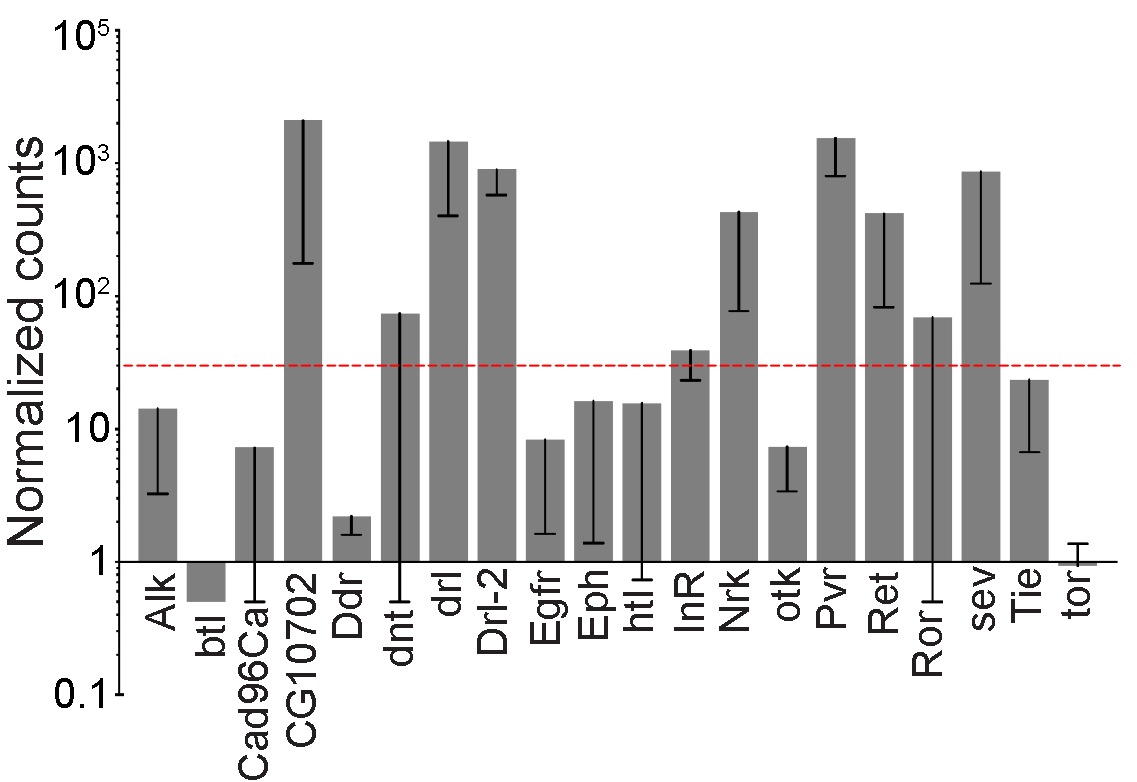

Supplement: S2 Fig — Transcriptomes were generated from microdissected ddaC soma after RNA isolation, cDNA conversion, and library preparation. RNA sequencing of 5′ skewed reads were mapped to transcript libraries and represent mRNA counts within neurons. Bars represent average transcript counts (log scale) and error bars SEM of 4 independent samples. A cutoff value was established (red segmented line) for screening of RTKs that might be important in neuronal processes. Quantitation is contained in S1 Data. da, dendritic arborization; ddaC, dorsal da C; RTK, receptor tyrosine kinase. (TIF) [file pbio.3000657.s002.tif]

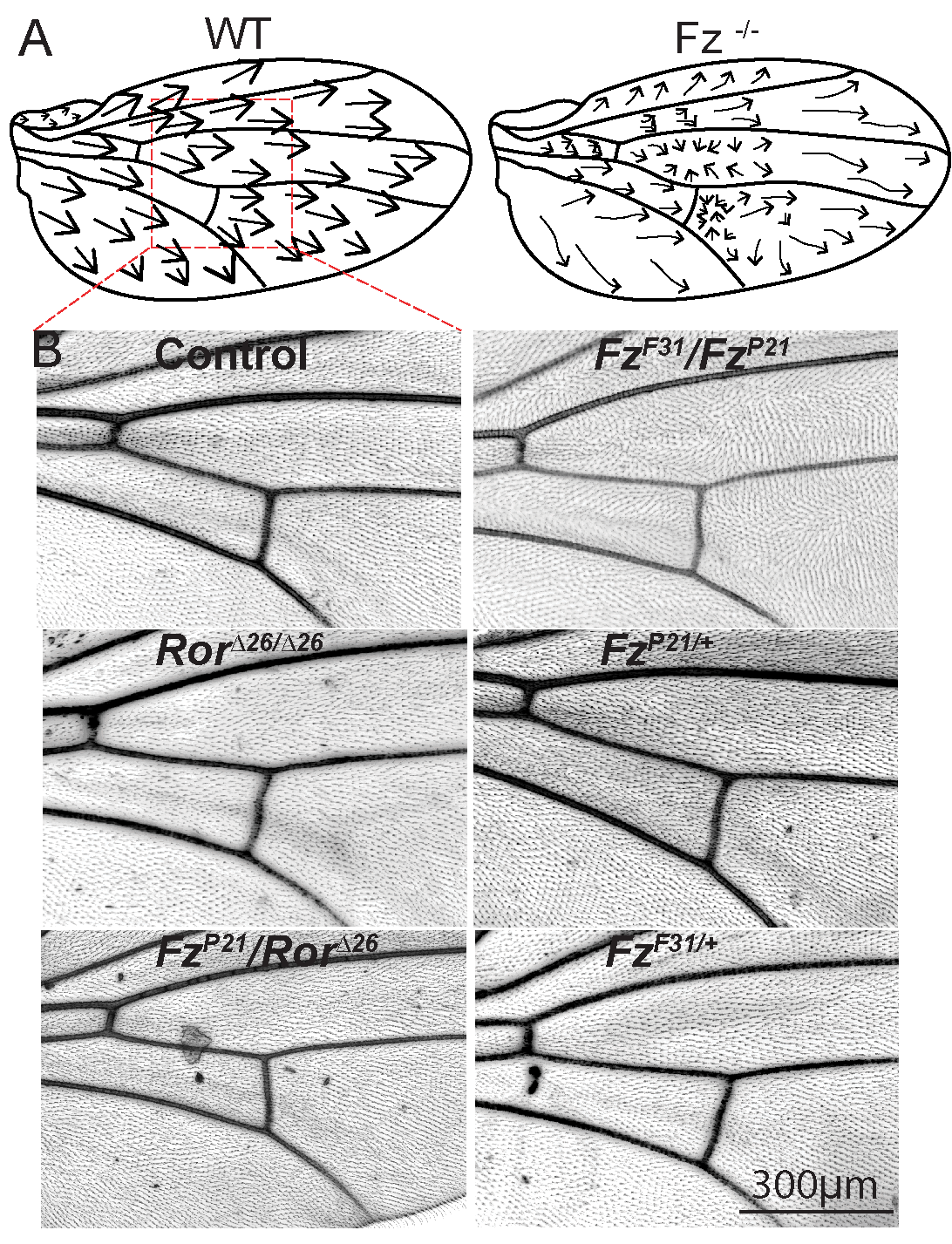

Supplement: S3 Fig — (A) Diagrams of normal (left) and perturbed (right) hair morphology on adult Drosophila wings. Wing hair organization is downstream of PCP signaling and is disrupted in strong fz loss-of-function mutants (B). Wing hair organization was assayed in Ror mutants and compared to fz mutants. 10–15 wings from adult male and female flies were imaged under high magnification (40×–100×, variable) on a Zeiss AxioZoom injection microscope under brightfield illumination. Images were acquired as z-stacks using CellSens software and processed in ImageJ and Adobe Photoshop by Focus Stacking for representative images to improve clarity. Blinded analyses could discern aberrant wing hair phenotypes only in strong fz loss-of-function animals (FzF31/P21). Ror mutants showed no discernable PCP phenotype. Quantitation is contained in S1 Data. fz, frizzled; PCP, planar cell polarity; Ror, RTK-like orphan receptor; RTK, receptor tyrosine kinase. (TIF) [file pbio.3000657.s003.tif]

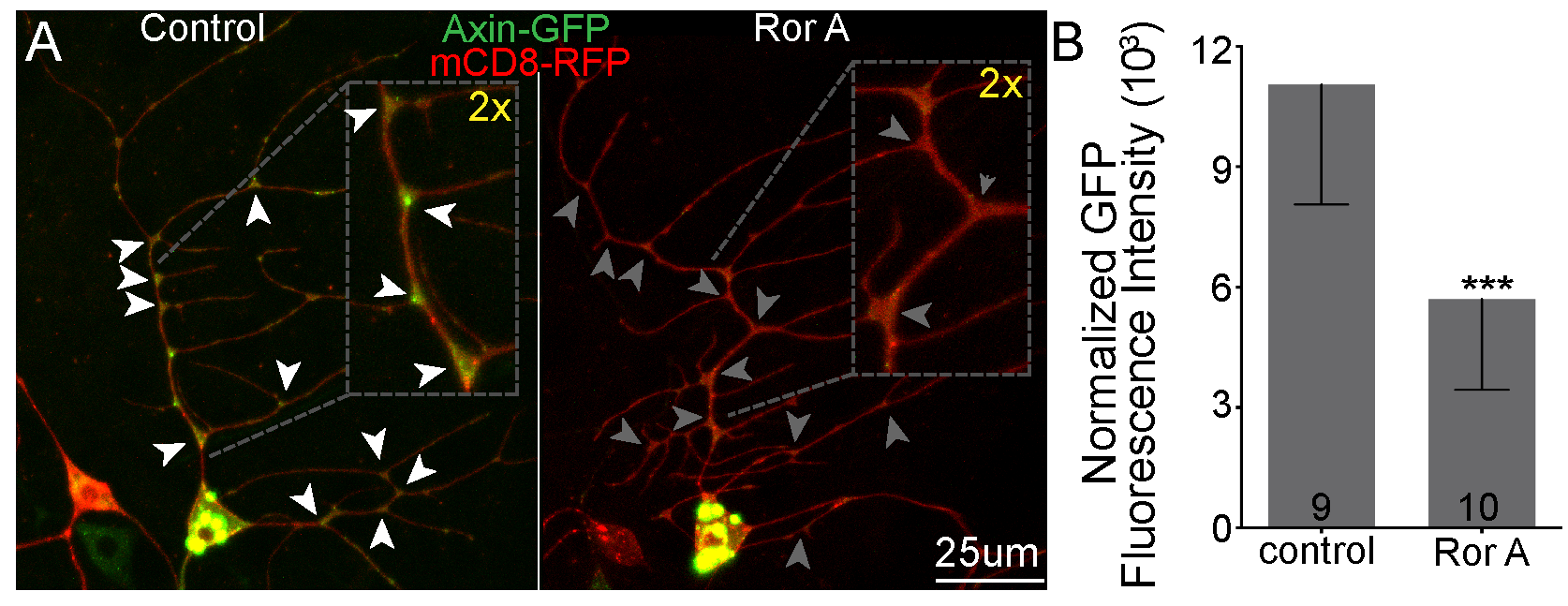

Supplement: S4 Fig — (A) Example images of ddaE neurons expressing UAS-Axin-GFP and UAS-mCD8-RFP. White arrows indicate BPs with normal Axin signal and gray arrows those with low signal. Insets show 2× magnification of indicated BPs. (B) Quantification of Axin-GFP fluorescence intensity was normalized as follows: BP − nBP = normalized FI. Multiple linear regression analysis; *P < 0.05, **P < 0.01, ***P < 0.001. Bars represent mean normalized FI ± SD; sample size (within bar, bottom) represents individual neurons/animals. Quantitation is contained in S1 Data. da, dendritic arborization; ddaE, dorsal da E; BP, branch point; FI, fluorescence intensity; GFP, green fluorescent protein; nBP, non-BP; RFP, red fluorescent protein; Ror, RTK-like orphan receptor; RTK, receptor tyrosine kinase; UAS, upstream activating sequence. (TIF) [file pbio.3000657.s004.tif]

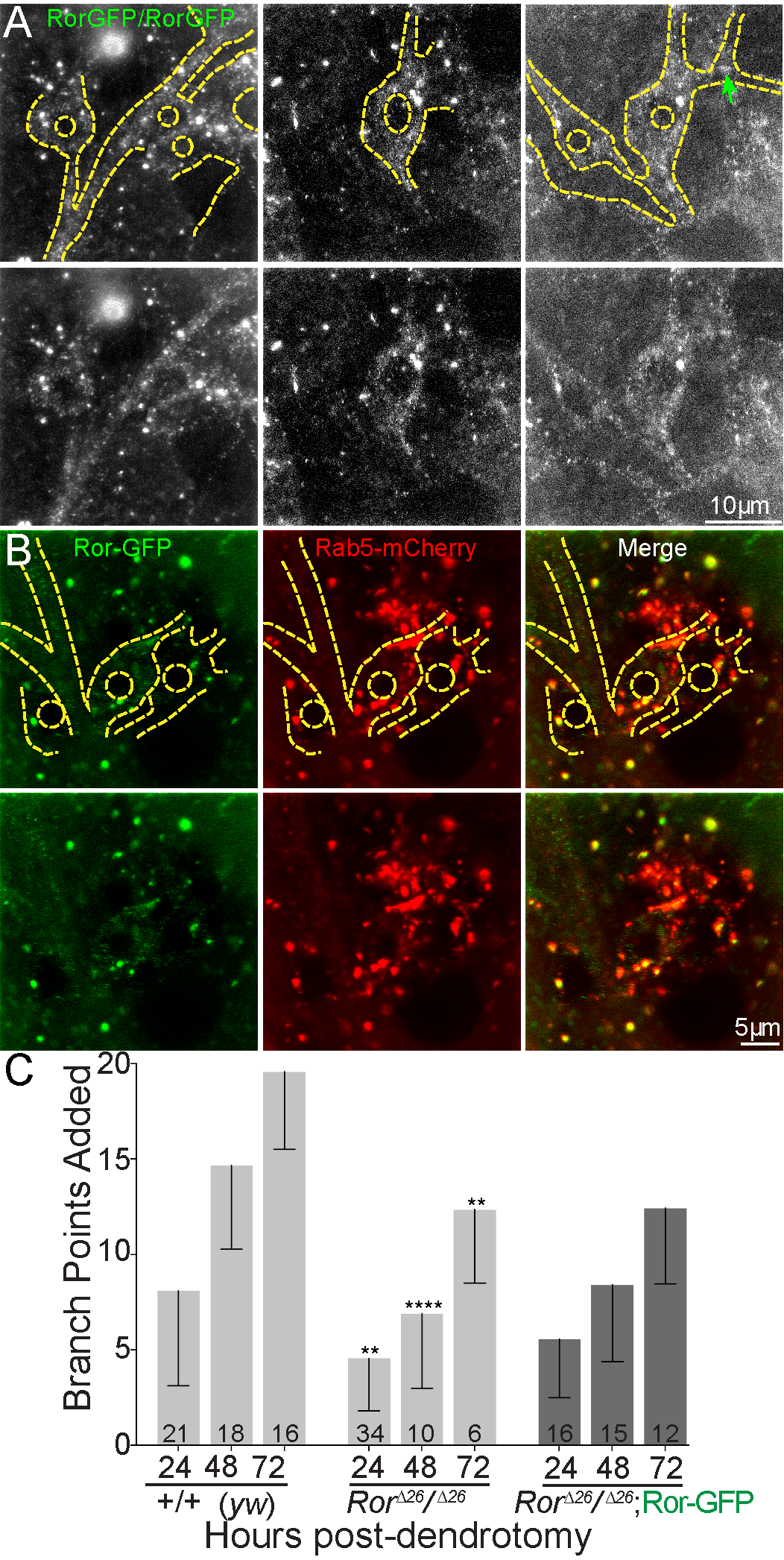

Supplement: S5 Fig — Transgenic Ror-GFP under control of the endogenous Ror promoter was visualized in larva expressing no other fluorophores (A) as homozygous Ror-GFP in a wild-type background. Weak Ror fluorescence can be discerned in soma, axons, and proximal dendrites of the dorsal arborization cluster. Animals with a single copy of the Ror-GFP allele show similar expression patterns as homozygous animals (B), and coexpression with transgenic Rab5-mCherry under its endogenous promoter shows potential colocalization in a subset of Rab5-labeled endosomes. However, we cannot rule out bleedthrough due to residual excitation of mCherry by 488-nm light. Images were acquired on an LSM800 inverted confocal microscope (63× oil plan-apo, 3× zoom) while anesthetized. (C) Dendrite regeneration in ddaE neurons homozygous for RorΔ26 and expressing transgenic Ror-GFP show impaired regeneration similar to homozygous mutants, suggesting transgenic Ror-GFP may be nonfunctional. Control and Ror mutant data are repeated from Fig 3 for comparison. Rescue experiment: RorΔ26/Δ26;221-mCD8-GFP crossed to RorΔ26/Δ26;Ror-p-Ror-GFP. Graphs show added branch points over 24, 48, and 72 h. Sample size (within bar, bottom) represents individual neurons from each animal. *P < 0.05, **P < 0.01, ***P P < 0.001 with Mann–Whitney U test to compare mean rank. Quantitation is contained in S1 Data. da, dendritic arborization; ddaE, dorsal da E; GFP, green fluorescent protein; Ror, RTK-like orphan receptor; RTK, receptor tyrosine kinase. (TIF) [file pbio.3000657.s005.tif]

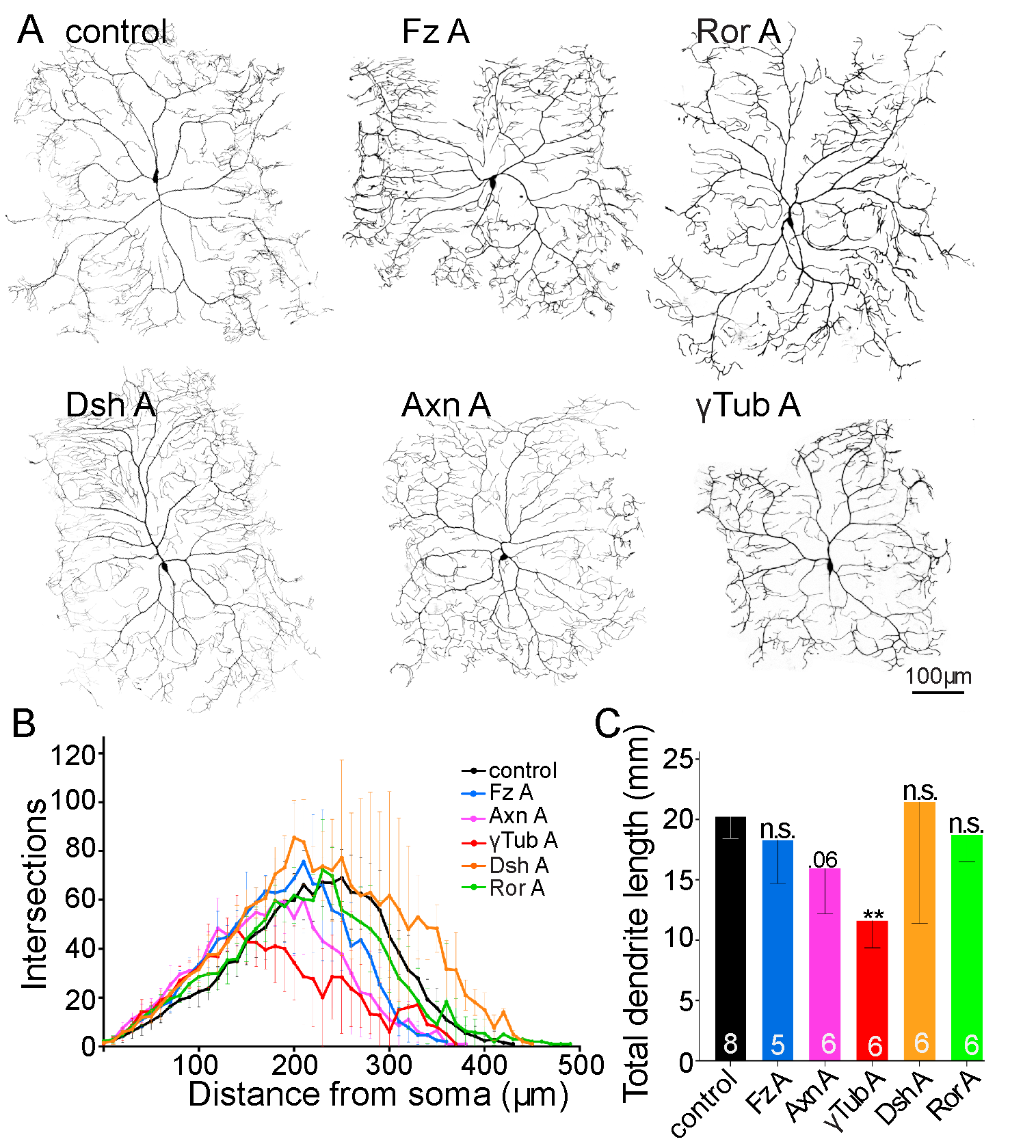

Supplement: S6 Fig — Class IV ddaC neurons expressing 1407-Gal4, ppk-Gal4, ppk-CD4-tdGFP;elav-Gal4, UAS-Dicer2, and respective UAS-RNAi hairpins were imaged in third-instar larva while anesthetized to create maximum intensity projections (A). Full arbor traces were obtained using the SNT plugin for ImageJ. Sholl analyses were acquired using the Sholl Analysis feature of SNT at 10-μm intervals (B). Total dendrite length (C) was compared using a Mann–Whitney U test to compare ranks. *P < 0.05, **P < 0.01, ***P < 0.001. Numbers on the bars are numbers of cells analyzed, and error bars are standard deviation. Quantitation is contained in S1 Data. da, dendritic arborization; ddaC, dorsal da C; GFP, green fluorescent protein; ppk, pickpocket; RNAi, RNA interference; SNT, Simple Neurite Tracer; tdGFP, tandem dimer GFP; UAS, upstream activating sequence. (TIF) [file pbio.3000657.s006.tif]

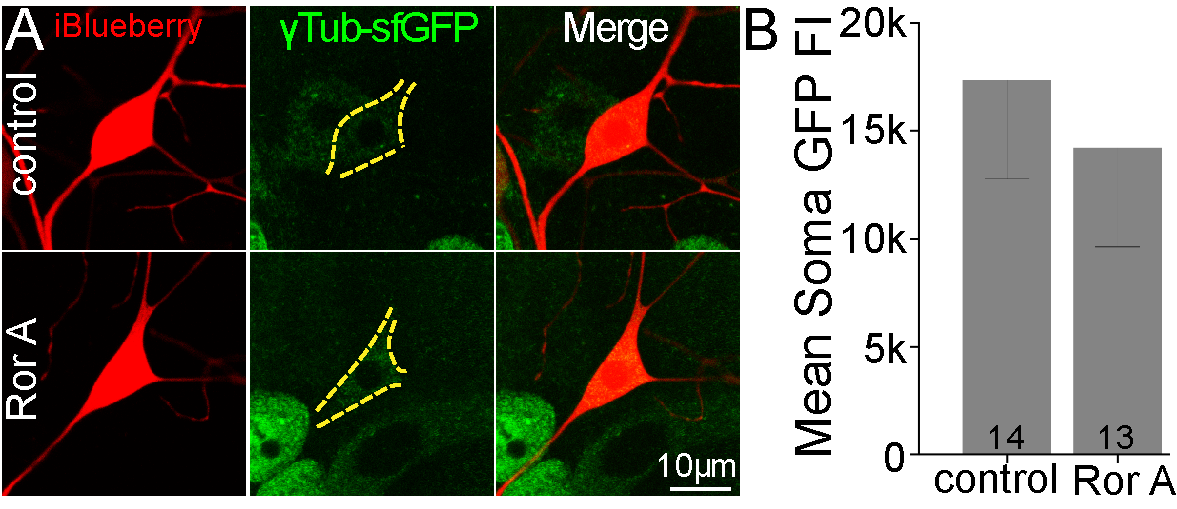

Supplement: S7 Fig — Class I neurons expressing a far-red fluorophore (UAS-iBlueberry, controlled by 221-Gal4) and endogenously tagged γTub-sfGFP and either control RNAi (A, top) or Ror RNAi 62868 (A, bottom). Total γTub levels were quantified as GFP fluorescence in a single optical slice from z-stacks acquired of the ddaE soma. Expression of endogenous γTub is too low to be reliably quantified in dendrites. Mean GFP FI was acquired in ImageJ, and levels (B) did not appreciably differ in Ror RNAi animals versus control. *P < 0.05, **P < 0.01, ***P < 0.001; Mann–Whitney U test. Numbers on the bars are numbers of cells analyzed, and error bars show standard deviation. Quantitation is contained in S1 Data. da, dendritic arborization; ddaE, dorsal da E; FI, fluorescence intensity; GFP, green fluorescent protein; RNAi, RNA interference; Ror, RTK-like orphan receptor; RTK, receptor tyrosine kinase; sfGFP, super-folder GFP; UAS, upstream activating sequence; γTub, γTubulin. (TIF) [file pbio.3000657.s007.tif]
