# Supplementary material for: The receptor tyrosine kinase Ror is required for dendrite regeneration in Drosophila neurons
Source: PLoS Biol. 2020 Mar 12;18(3):e3000657. doi: 10.1371/journal.pbio.3000657 (PMC7067388; doi:10.1371/journal.pbio.3000657)
Supplement: S1 Supplemental Methods — (DOCX) [file pbio.3000657.s010.docx]

**Supplemental methods**

***Wing hair analysis***

*Drosophila* wings were dissected from adult flies of respective genotypes and immediately imaged under brightfield magnification (Zeiss AxioZoom). Image files (CellSens software) were processed using ImageJ and Adobe Photoshop to improve clarity and contrast. Experiments show representative images from 10-20 wings (5-10 flies) of corresponding genotypes and equal male/female distribution.

***Imaging of ddaC dendrite arbors expressing RNAi hairpins***

Tester line virgin female flies expressing 1407-Gal4, ppk-Gal4, ppk-CD4-tdGFP;elav-Gal4, UAS-Dicer2 were crossed to male flies expressing respective UAS-RNAi hairpins to knockdown gene expression pan-neuronally while labeling only class IV ddaC neurons. Food caps containing embryos were collected every 24 hours and aged 3 days at 25C. Third instar larva were briefly anesthetized for 2 minutes using isoflurane in an enclosed chamber as described previously [1]. Larva were mounted on a microscope slide with a dried agarose pad and secured with a cover slip and tape.

***Laser capture microdissection of ddaC neurons***

L3 larva with ddaC neurons expressing mCD8-GFP under ppk-Gal4 control were prepared for cryosectioning by placing spot-dried larva in cryomatrix (Thermo-Fisher cat 6769006). After placing larva in cryomatrix, the mold was cooled on ice to slow larva movement and obtain straight larva for the mold. Samples were then snap frozen on a block of dry ice and stored at -80°C. Larva were sectioned on a Leica CM1900 cryostat. Chamber temperature was set to -24°C, with block temperature set to approximately -26°C. Head angle was set between 2-5° and 8um sections were placed directly onto Carl Zeiss PEM membrane slides (415190-9041-000) that had been exposed to UV light for 30min to improve hydrophilicity. Cryosections could be stored at -80°C long term if needed. To prepare cryosections for laser capture microdissection, slides were moved through the following solutions on ice to remove cryomatrix and dehydrate the samples: 70% ethanol, nuclease-free water, 70% ethanol, 85% ethanol, 100% ethanol, and a separate 100% ethanol. Slides were allowed to dry for ~10 minutes prior to laser capture microdissection. Laser capture microdissection was performed on a Carl Zeiss PALM laser capture microdissection system. 10 ddaC cells were captured into a single adhesive cap (Carl Zeiss item number 415190-9191-000).

***Generation of cDNA libraries from microdissected neurons***

cDNA libraries were prepared directly from ten microdissected ddaC neurons according to the following protocol adapted from previous studies [2, 3]. 16ul of digest buffer was applied directly to the cap, and tubes were incubated at 42°C for 1h. Following incubation, samples were spun at 2,500g at room temperature. 4ul of freshly prepared stop buffer were added to samples. 18ul were transferred into a PCR tube and placed on ice. 2ul of SuperScript III reverse transcriptase was added and thermocycler protocol #1 was ran. At the first pause step, samples were placed on ice and 4ul of RNaseH-Mg2+ mixture was added to the sample. Sample was continued on thermocycler protocol #1. At the second pause, 14ul of tailing buffer and 0.8ul terminal transferase were added and protocol #1 was then finished. Following completion of protocol #1 the thermopol reaction was assembled. This was amplified according to thermocycler protocol #2 with samples split into 3 separate tubes for the first 25 cycles. Following completion of cycle25, samples were re-pooled into a single tube for the final 5 amplification cycles.

Digest buffer:

5x first-strand buffer: 20 μl

1x stock primer mix: 2 μl

20 mg/ml proteinase K: 1 μl

Nuclease-free water: 57 μl

Total volume: 80 μl
Primer mix: Prepare 25X stock by adding 15ul nuclease-free water, 5um of 100mM dATP, 5ul of

100mM dGTP, 100mM dTTP, 100mM dCTP, 5ul of 80 OD/mL olido(dT)24 primer.

Digest stop buffer

20 U/ml anti-RNase: 1 μl

20 U/ml SUPERase-In: 1 μl

100 mM PMSF: 1 μl

Nuclease-free water: 17 μl

Total volume: 20 μl
Thermopol reaction

Thermopol buffer: 10 μl

100 mM MgSO4: 2.5 μl

20 mg/mL BSA: 0.5 μl

10 mM dNTP: 10 μl

Taq: 2 μl

AL1 primer*: 0.3 μl

nuclease free H2O: 35.6 μl

Sample: 38.8 μl

Total: 100 μl

Thermocycler protocol #1

Temperature Time

50°C, 15 min

70°C, 15 min

Pause (see step 9) Press Enter

37°C, 1 min*

Pause (see step 10) Press Enter

37°C, 15 min

Pause (see step 12) Press Enter

37°C, 15 min

65°C, 10 min

4°C, Hold

Thermocycler protocol #2. Samples are re-pooled after cycle 25

| Cycle | Denaturation | Annealing | Extension |
| --- | --- | --- | --- |
| 1-4 | 94°C for 1 min | 32°C for 2 min | 72°C for 6 min with 10s increase each cycle |
| 5-25 | 94°C for 1 min | 42°C for 2 min | 72°C for 6 min with 40s increase each cycle |
| 26-30 | 94°C for 1 min | 42°C for 2 min | 72°C for 6 min |

Part numbers: First strand buffer and Superscript III enzyme (Invitrogen cat. No. 18080-044); proteinase-K (Sigma-Aldrich cat. No. P2308); nuclease-free water (Ambion cat. No. AM9932); anti-RNAse (Ambion, at. No AM2690); SUPERase-In (Ambion, at. No AM2694); PMSF (Sigma-Aldrich cat. No. P7626); dNTP set (Roche cat. no. 11277049001); RNAse H (New England Biolabs, cat. No. M097L); BSA (Roche, cat. no. 10711454001); Terminal transferase buffer (Invitrogen, cat. no. 16314-015); terminal transferase (Roceh, cat. no. 03333574001); ThermoPol buffer (New England Biolabs, cat. no. B9005S) AL1 primer sequence: 5’-

ATTGGATCCAGGCCGCTCTGGACAAAATATGAATCTTTTTTTTTTTTTTTTTTTTTTTT-

3’

***RNA sequencing***

RNAseq libraries were prepared using Illumina Nextera Reagents as described [4]. Sequencing was performed on an Illumina HiSeq instrument with 150bp single end reads.

***Bioinformatics***

The following bioinformatics workflow was used:

FASTQC – quality control

Trimmomatic – adapter and quality trimming

Tophat – alignment to Drosophila genome (flybase release dmel6.04)

Cufflinks43–45 – DE analysis

**References**

1. Poe AR, Tang L, Wang B, Li Y, Sapar ML, Han C. Dendritic space-filling requires a neuronal type-specific extracellular permissive signal in Drosophila. Proceedings of the National Academy of Sciences of the United States of America. 2017;114(38):E8062-E71. doi: 10.1073/pnas.1707467114. PubMed PMID: 28874572; PubMed Central PMCID: PMCPMC5617288.

2. Wang L, Janes KA. Stochastic profiling of transcriptional regulatory heterogeneities in tissues, tumors and cultured cells. Nat Protoc. 2013;8(2):282-301. doi: 10.1038/nprot.2012.158. PubMed PMID: 23306461; PubMed Central PMCID: PMCPMC3818581.

3. Janes KA, Wang CC, Holmberg KJ, Cabral K, Brugge JS. Identifying single-cell molecular programs by stochastic profiling. Nature methods. 2010;7(4):311-7. Epub 2010/03/17. doi: 10.1038/nmeth.1442. PubMed PMID: 20228812; PubMed Central PMCID: PMC2849806.

4. Singh S, Wang L, Schaff DL, Sutcliffe MD, Koeppel AF, Kim J, et al. In situ 10-cell RNA sequencing in tissue and tumor biopsy samples. Sci Rep. 2019;9(1):4836. doi: 10.1038/s41598-019-41235-9. PubMed PMID: 30894605; PubMed Central PMCID: PMCPMC6426952.
